# Regulatory Pathway Analysis by High-Throughput In Situ Hybridization 

**DOI:** 10.1371/journal.pgen.0030178

**Published:** 2007-10-19

**Authors:** Axel Visel, James Carson, Judit Oldekamp, Marei Warnecke, Vladimira Jakubcakova, Xunlei Zhou, Chad A Shaw, Gonzalo Alvarez-Bolado, Gregor Eichele

**Affiliations:** 1 Department of Genes and Behavior, Max Planck Institute of Biophysical Chemistry, Goettingen, Germany; 2 Genomics Division, Lawrence Berkeley National Laboratory, Berkeley, California, United States of America; 3 Biological Monitoring and Modeling Department, Pacific Northwest National Laboratory, Richland, Washington, United States of America; 4 Department of Molecular and Human Genetics, Baylor College of Medicine, Houston, Texas, United States of America; Harvard Medical School, United States of America

## Abstract

Automated in situ hybridization enables the construction of comprehensive atlases of gene expression patterns in mammals. Such atlases can become Web-searchable digital expression maps of individual genes and thus offer an entryway to elucidate genetic interactions and signaling pathways. Towards this end, an atlas housing ∼1,000 spatial gene expression patterns of the midgestation mouse embryo was generated. Patterns were textually annotated using a controlled vocabulary comprising >90 anatomical features. Hierarchical clustering of annotations was carried out using distance scores calculated from the similarity between pairs of patterns across all anatomical structures. This process ordered hundreds of complex expression patterns into a matrix that reflects the embryonic architecture and the relatedness of patterns of expression. Clustering yielded 12 distinct groups of expression patterns. Because of the similarity of expression patterns within a group, members of each group may be components of regulatory cascades. We focused on the group containing Pax6, an evolutionary conserved transcriptional master mediator of development. Seventeen of the 82 genes in this group showed a change of expression in the developing neocortex of *Pax6*-deficient embryos. Electromobility shift assays were used to test for the presence of Pax6-paired domain binding sites. This led to the identification of 12 genes not previously known as potential targets of Pax6 regulation. These findings suggest that cluster analysis of annotated gene expression patterns obtained by automated in situ hybridization is a novel approach for identifying components of signaling cascades.

## Author Summary

Signaling pathways drive biological processes with high specificity. Reductionist approaches such as mutagenesis provide one strategy to identity components of pathways. We used high throughput in situ hybridization to systematically map the spatiotemporal expression pattern of ∼1,000 developmental genes in the mouse embryo. The rich information collectively contained in these patterns was captured in annotation tables that were systematically mined using hierarchical clustering, resulting in 12 groups of genes with related expression patterns. We show that this process generates biologically meaningful, high-content information. The expression pattern of developmental master regulator Pax6 is found in a cluster together with that of 81 other genes. The paired DNA binding domain of Pax6 can bind to regulatory sequences in 14 of the 81 genes. We also found that the expression pattern of all these 14 genes is up- or downregulated in Pax6 mutant mice. These results emphasize that determining the expression pattern of many genes in a systematic way followed by an application of integrative tools leads to the identification of novel candidate components of signaling pathways. More generally, when complemented with appropriate data-mining strategies, transcriptome-scale in situ hybridization can be turned into a powerful instrument for systems biology.

## Introduction

Basic processes that cells go through—fate specification, proliferation, differentiation, migration, and programmed death—are driven by gene networks that are, for the most part, still poorly understood. In the past few years, several large-scale approaches have been launched to begin unraveling the regulatory pathways governing cell behavior. Novel strategies include RNA interference screens, interactome analysis, transcriptome mapping by microarrays, and ChIP-on-chip assays. Such cell-based analyses portray regulatory pathways as complex networks [1]. Analysis of gene expression by in situ hybridization (ISH) has proven to be a powerful means to validate pathways because ISH provides high-resolution information on gene expression in cells within the context of location in the organ or organism [2]. Recently, ISH on tissue sections has been automated, making it possible to determine gene expression patterns for thousands of genes, and thus enabling the construction of gene expression atlases (e.g., [3–5]; reviewed by [6]).

**Figure 1:**
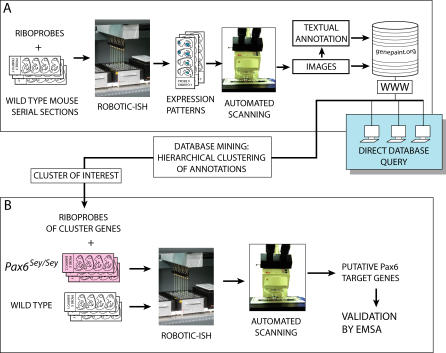
Building and Mining the Genepaint.org Expression Pattern Atlas (A) Robotic ISH uses digoxigenin-tagged riboprobes that are hybridized to serial sections of wild-type mouse embryos. Images of expression patterns are captured by automated microscopy, annotated, and deposited on the genepaint.org database. Data can be queried using standard web browsers. (B) In the present study, expression pattern atlas data were subjected to hierarchical clustering and genes that grouped with Pax6 were passed through several “filters” for validation purposes including ISH with *Pax6^sey^* loss-of-function embryos and EMSAs to detect PAX6-paired domain binding sites.

In view of past success using ISH data in the validation of single regulatory interactions, it is likely that transcriptome-scale gene expression atlases will facilitate large-scale validation of regulatory networks [7] and, more importantly, contribute to the discovery of network components. To provide a proof of concept for this idea, we first applied ISH to generate a gene expression atlas of the midgestation mouse embryo (E14.5) populated with approximately 1,000 genes, including known developmental genes as well as many genes whose expression and function in development has not previously been examined. We then textually annotated expression patterns of this atlas and utilized hierarchical clustering to mine for genes involved in the development of the cerebral neocortex, a brain region that has undergone dramatic structural and functional enhancement during mammalian evolution. Thereafter, we validated candidate genes by a combination of biochemical assays and ISH analyses on mutant embryos (Figure 1).

The present study focuses on genes regulated by Pax6, a transcription factor with both a paired domain and a homeodomain. Pax6 is an evolutionary highly conserved master mediator of development [8] and plays important roles in the formation of the mammalian cerebral cortex [9], eye [10], and pancreas [11,12]. The cortex of the midgestation mouse embryo consists of the ventricular, subventricular, and intermediate zones (VZ, SVZ, and IZ, respectively), a subplate, a cortical plate, and the MZ (marginal zone) [9]. *Pax6* is expressed in the VZ and SVZ where the stem cells of the cortex reside. A naturally occurring *Pax6* null mutation termed Small eye (*Pax6^sey^* or *sey*) [13] is characterized by an enlargement of VZ and SVZ and an IZ that is populated by neurons normally found in the SVZ [14,15]. At the molecular level, *Pax6* and the nuclear orphan receptor gene *Nr2e1* (*Tlx*) appear to synergistically regulate the formation of the SVZ in which progenitors of the outer cortical layers arise (reviewed by [16]). Hence in the absence of *Pax6* and even more so in the absence of both *Pax6* and *Nr2e1*, the outer cortical layers are severely affected [17]. Our computational analysis of annotated expression patterns generated by large-scale ISH led to 12 new genes harboring a Pax6-paired domain binding site. More generally, this gene expression atlas of the midgestation embryo combined with robotic ISH offers a novel approach for finding putative components of genetic networks regulating critical aspects of mammalian development.

## Results

### The 1K ISH Dataset

The spatial expression patterns of 1,030 genes (“1K dataset”, Table S1) were determined by ISH on series of 24 equally spaced sagittal tissue slices from E14.5 embryos. This stack of sections provides a comprehensive representation of the tissues and organ primordia characteristic for this developmental stage. Digoxigenin-tagged riboprobes used for ISH were prepared by in vitro transcription from DNA templates that had been generated by PCR from cDNA using pairs of gene-specific primers (for details see [18]). Following robotic ISH, high-resolution images of all sections were captured using a custom-made automated microscope [19] and resulting images were deposited on a public database, termed “genepaint.org” (Figure 1A and [20]). Images are retrievable from this database using gene symbol, gene name, gene ID, or genepaint set ID [20], which are provided in Table S1. Images can be viewed using browser-integrated applications.

A total of 887 (86%) genes of the 1K dataset were at least weakly expressed at E14.5 while 144 (14%) transcripts were not detected in any tissue. In parallel to the ISH analysis, a global transcriptome analysis of E14.5 embryos was carried out using Affymetrix 430 2.0 chips. These data have been made publicly available through the National Center for Biotechnology Information Gene Expression Omnibus (NCBI GEO) database (see database accession number below). Using the absolute call attribute “present” as indication for expression, ∼13,000 genes were considered to be transcribed at E14.5. Of the 887 genes that were found to be expressed by ISH, 757 could be unambiguously linked to one or several probe sets on the microarray. Of these, 480 (63%) were scored as present by at least one probe set on each duplicate array. The discrepancy between ISH and microarray is partly due to transcripts that are restricted to a small number of cells (see [18] for a discussion).

**Figure 2:**
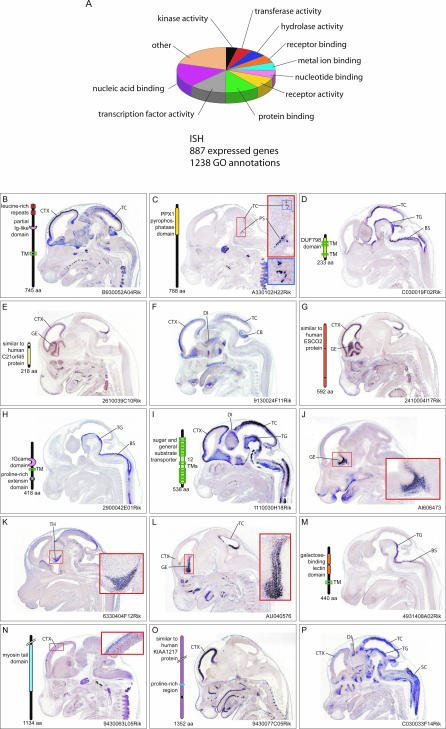
Examples of Gene Expression Patterns in E14.5 Mouse Embryos (A) Pie chart shows the distribution of the ten most abundant molecular function classes of the 887 expressed genes of the 1K dataset. (B–P) Sample expression patterns of genes with highly regionalized brain expression at E14.5. Shown are sagittal sections through the head. Typical examples of regional expression in the cortex (ctx) are seen in (B, E–G, I, N, and O), where transcripts are either found in the ventricular layer (E, G, O) or in the MZ (B, F, I, N; see Figure 7A for layer definitions). Scattered expression is illustrated by the insets of (C) where single *A330102H22Rik*-positive cells can be seen. Diagrams on the left illustrate predicted open reading frames and their putative domain structures. Abbreviations: BS, brain stem; CB, cerebellum; CTX, cortex; DI, diencephalon; GE, ganglionic eminence; PS, pons; SC, spinal cord; TC, tectum; TG, tegmentum; TH, thalamus.

Expert analysis of the ISH data (see below) showed that 74% (652) of the 887 transcribed genes were expressed in a regional manner, i.e., transcripts for a particular gene were either distributed in a nonuniform manner within a specific tissue or found only in a subset of tissues and organs (Figure 2A). Typical examples for regionally expressed genes directly downloaded from genepaint.org are shown in Figure 2B–2P. Note that robot-generated ISH data have sufficient quality and resolution to unambiguously tie expression to specific anatomical structures occasionally as minute as a single cell (e.g., Figure 2C inset).

### Annotation of Expression Patterns

**Figure 3:**
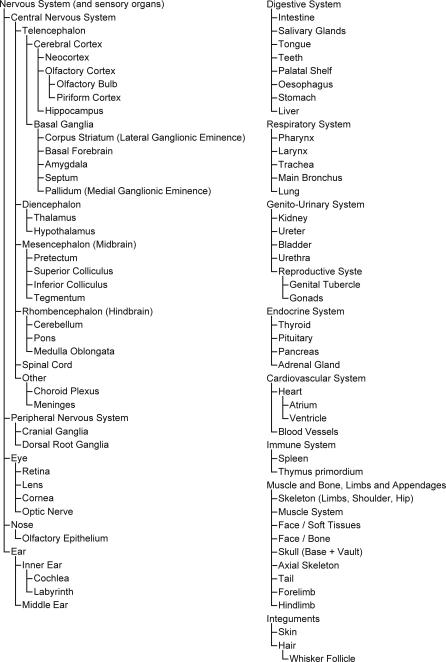
Tree of Anatomical Structures That Were Annotated for Gene Expression For details of annotation methods and terminology, see “Annotation of Expression Patterns” in Results.

To enable searches for expression patterns in genepaint.org and to compare patterns across a large number of genes, we implemented an expert-based controlled vocabulary annotation of expression patterns. A total of 96 hierarchically organized structures (Figure 3) were annotated, which collectively represent the majority of organ primordia, tissues, and tissue subregions that characterize the E14.5 embryo. Although the hierarchical tree is composed of 96 items, only 70 structures are unique “leaves” of the tree, i.e., do not overlap with or fully encompass other structures. For each of the 96 structures, two attributes were allocated. The first attribute indicates the type of expression pattern characteristic for the structure in question (regional, scattered, and ubiquitous; see Figure 2B–2P for examples). The second attribute is the intensity of expression (not detected, low, medium, and strong expression; see also [20] for definition of attributes). In all cases, each annotation was determined by one annotator and confirmed independently by a second expert. In the rare cases of a discrepancy, a consensus decision was found. These textual annotations for all 96 structures for each of the 652 regionally expressed genes are available on genepaint.org, thus enabling user-initiated searches for genes that are expressed with a particular pattern and strength in a single or in multiple structures (for instructions to carry out such a search using the “structure selection tool” see “Quickguide” of genepaint.org). For example, searching for genes that are regionally and strongly expressed in the neocortex of an E14.5 embryo will produce a list of ∼170 genes (August 2007). The validity of queries can readily be checked visually by using the image viewing applications integrated in genepaint.org.

### Computational Analysis of Gene Expression Pattern Annotation

Annotation of expression patterns across anatomical structures enables hierarchical clustering of patterns with the prospect of revealing similarities between expression patterns. Within the set of 652 patterns, for every possible pair of pattern annotations, a distance score was calculated to measure the similarity between the two patterns across all anatomical structures. The scoring function at each structure was derived from both the strength of gene expression and the pattern. Genes were hierarchically clustered based upon these scores. Using an analogous procedure, the distances between all pairs of anatomical structures were calculated on the basis of the annotated patterns of expression across all genes. These latter scores were utilized to cluster the anatomical regions (for details see Text S1).

**Figure 4:**
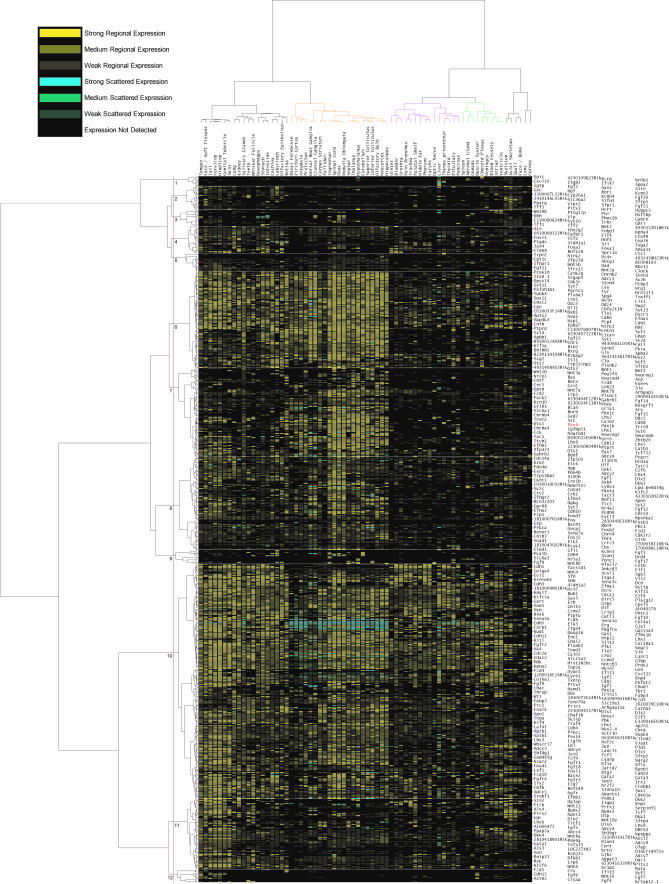
Cluster Analysis of Expression Pattern Annotations The annotation of the expression pattern for each gene at every anatomical location is represented by color (yellow for regional or ubiquitous, blue for scattered) and intensity (dark for weak expression, midtone for medium expression, and bright for strong expression). See also color key on figure. Dendrograms composed using Ward's clustering represent 70 unique anatomical structures (horizontal) and 652 genes (vertical). A total of 12 clusters/groups of genes are labeled.

Figure 4 shows the result of this cluster analysis, which is characterized by two main features. First, tissues that have similar embryonic origin and/or similar physiological functions group together (Figure 4, dendrogram on top). For example, all components of the central and peripheral nervous systems cluster together (red branches in dendrogram). Likewise, endoderm-derived endocrine organs (pancreas, thyroid, pituitary, and liver) group together, and organs of the vascular system (choroids plexus, atrium, ventricle, blood vessels, and meninges) have a shared expression pattern and thus group. Organs equipped with sensory epithelia (retina, cochlea, labyrinth, and olfactory epithelium) as well as organs containing polarized epithelia (lung, kidney, salivary glands, oesophagus, intestine, and stomach) form groups. Thus, hierarchical clustering of expression patterns unravels relationships between tissues and to a significant extent the dendrogram recapitulates both the developmental lineage relationships and the shared physiological functions. The second feature of Figure 4 is the classification of the 652 regional expression patterns into 12 groups. Groups 1–5, 9, and 11 include genes expressed in a limited number of tissues, while group 10 genes are more broadly expressed. Groups 7 (82 genes) and 8 (86 genes) represent transcripts with a bias to the nervous system. Hierarchical clustering thus systematically compares hundreds of complex expression patterns to group related structures in a way reflecting the body plan of the organism. The emergence of previously established relationships in the embryonic architecture from a statistical analysis of controlled vocabulary annotation validates in part the second result of clustering—the presence of groups of similarly expressed genes.

**Figure 5:**
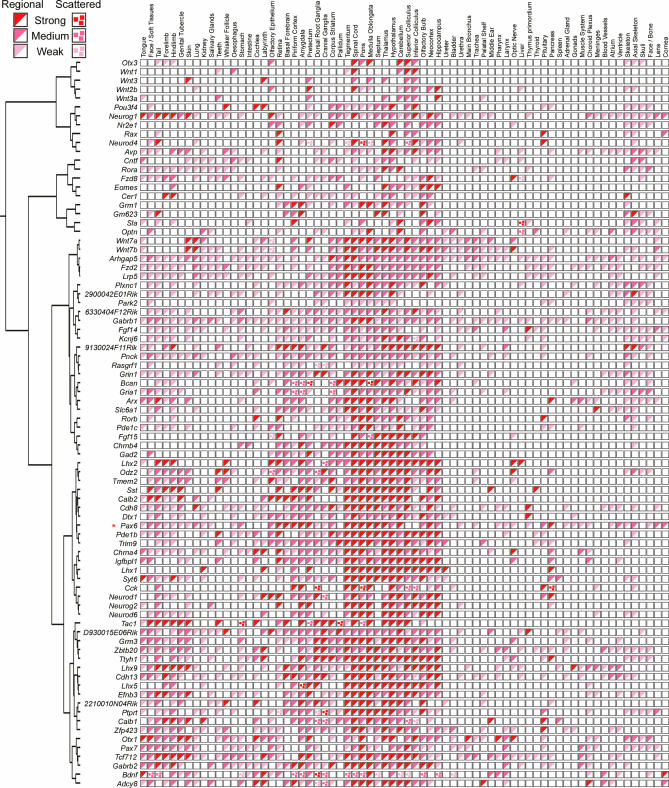
Detailed View of Cluster 7 with Pattern and Annotation Icons Used in Genepaint.org Notice the predominance in this cluster of genes with “strong regional” expression in the central nervous system. Icons are defined in the inset on the top left.

**Figure 6:**
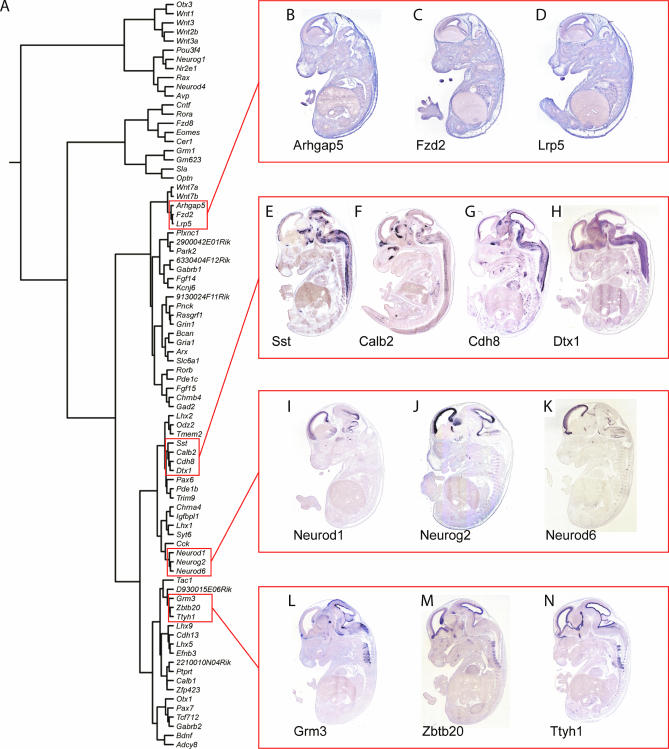
Hierarchical Clustering Is Consistent with Image Data (A–N) Selected expression patterns of genes from cluster 7 showing that genes located at the same branch of the tree have similar expression patterns.

To better appreciate how the spatial expression pattern affects grouping of genes, in Figure 5 we show cluster 7 at higher magnification using the full spectrum of genepaint pattern and intensity annotation icons. The dominance of expression of cluster 7 genes in the tissues of neuroectodermal origin is evident, albeit the genes in the top portion of the tree (*Otx3* to *Lrp5*) are also significantly expressed in mesoderm-derived tissues. For example, the group consisting of *Arhgap5*, *Fzd2*, and *Lrp5* are widely expressed in cells of mesodermal origin but still show expression in the VZ of the telencephalon (Figure 6, top red box). Figure 6 additionally illustrates the point that genes found on distinct short branches of the tree have nearly identical expression patterns (see pictures of embryos enclosed in red boxes of Figure 6). For example, a metabotropic glutamate receptor (*Grm3*), a zinc finger-containing transcription factor (*Zbtb20*), and a putative chloride channel (*Ttyh1*) share a regional expression pattern in the ventral part of the telencephalon, in midbrain, hindbrain, spinal cord, and in dorsal root ganglia (red box at the bottom of Figure 6). It thus appears that the clustering was successful in arranging annotated patterns of expression in a meaningful way that is consistent with the image data.

### Pax6-Candidate Target Identification Using the Genepaint.org Database

**Figure 7:**
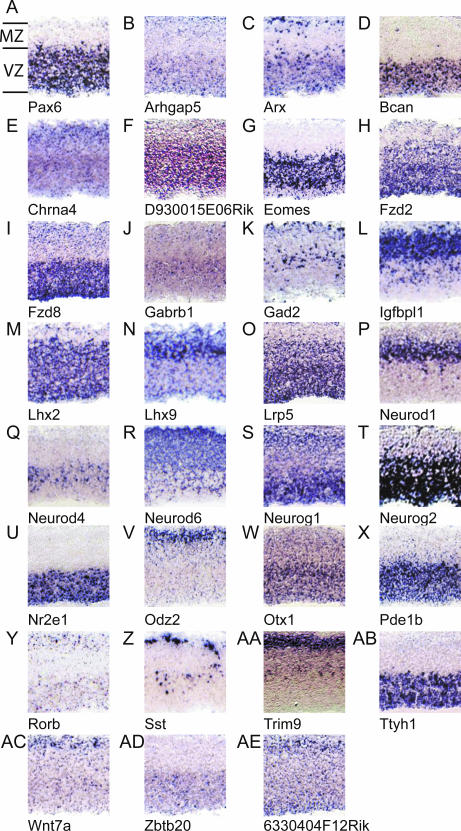
Expression Pattern of 30 Genes That Are in Cluster 7 but Are also Coexpressed with Pax6 in the Cortex of the E14.5 Mouse Embryo (A–AE) *Pax6* is strongly expressed in the VZ, and for the majority of the other genes this is also the case. Note however, genes such as *Sst* or *Trim9* are expressed in a subset of VZ cells and additionally are also expressed in the MZ.

Genes with similar expression patterns may be part of a common signalling pathway. Many of the 82 genes of group 7 (Figures 4–6 and first column in Table S2) encode proteins involved in signal transduction including Pax6, which plays a key role in multiple developmental processes of the nervous system (see Introduction and Discussion). Theoretically, among the 82 genes, several could be up- or downstream of Pax6. Because the clustering matrix is derived from gene expression in the entire spectrum of annotated E14.5 embryonic tissues, we do not expect all 82 genes of group 7 to be coexpressed with Pax6 in the VZ of the developing cortex. To clarify this point, expression of all 82 genes in the germinal zone was examined in the appropriate E14.5 datasets of genepaint.org (Figure 7) revealing that 40% (*n* = 30) were coexpressed with Pax6 and are thus candidates for either regulating or being regulated by Pax6 (column 2 of Table S2).

### Altered Expression of Putative Pax6 Targets in Pax6^sey/sey^ Embryos

To determine which of the 30 genes coexpressed with Pax6 in the VZ of the E14.5 cortex are potentially part of a Pax6 regulatory network, we passed the candidates through two additional “filters” (Figure 1B). First, we determined expression patterns in Pax6-deficient embryos (Pax6^sey/sey^), as it is expected that the pattern of expression of Pax6 downstream genes would be changed in mutant tissue. Second, we searched for and subsequently experimentally validated Pax6-paired domain binding sites in those genes whose expression pattern was augmented or decreased in Pax6^sey/sey^ cortex.

**Figure 8:**
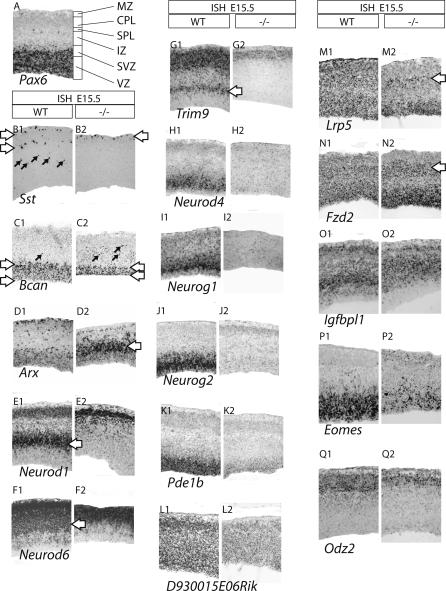
Expression Pattern of Pax6 Candidate Targets in the Cortex of E15.5 *Pax6^sey/sey^* Embryos (A–Q) The majority of genes are downregulated in mutant cortex. For details, see “Altered Expression of Putative Pax6 Targets in *Pax6^sey/sey^* Embryos” in Results. Abbreviations: CPL, cortical plate; IZ, intermediate zone; MZ, marginal zone; SPL, subplate; SVZ, subventricular zone; VZ, ventricular zone; WT, wild type; -/-, *Pax6^sey/sey^* mutant.

To examine the expression pattern of the 30 candidate genes, we applied robotic ISH to sections of E15.5 mutant and wild-type embryos (Figure 1B). At this developmental stage, cortical layering becomes very distinct (Figure 8A) and hence the Pax6^sey/sey^ cortical phenotype is clearly noticeable. Comparing ISH results of wild-type and mutant cortices (Figure 8) yielded a total of 16 genes that have an altered expression pattern in the developing cortex of Pax6^sey/sey^ mice (Table S2, third column). These genes are *Arx*, *Bcan*, *D930015E06Rik*, *Eomes*, *Igfbpl1*, *Fzd2*, *Lrp5*, *Neurod1*, *Neurod4*, *Neurod6*, *Neurog1*, *Neurog2*, *Odz2*, *Pde1b*, *Sst*, and *Trim9*. Several ISH results shown in Figure 8 were validated by quantitative real-time PCR with the result that ISH and quantitative PCR (qPCR) analyses are consistent (Figure S1). Nevertheless, ISH provides a more detailed portrait than qPCR of how the absence of Pax6 protein affects gene expression. For example, while qPCR data indicate that Neurod1 is significantly downregulated in mutant cortex, ISH shows that this downregulation occurs predominantly in the IZ. In the case of Trim9, qPCR indicates a 2-fold reduction in overall expression level. The ISH data attribute this reduction to the loss of a band of expression in the SVZ. The upregulation of Arx predicted by qPCR is focused to the SVZ.

Inspection of the cortical expression patterns of the 16 candidates allowed us to classify them into two groups. Some genes show qualitative changes of expression pattern in the mutant cortex, while others show a mostly quantitative change over the entire area of expression. Genes belonging to the first group are *Sst* (Figure 8B1 and 8B2), *Arx* (Figure 8D1 and 8D2), *Neurod1* (Figure 8E1 and 8E2), *Neurod6* (Figure 8F1 and 8F2), and *Trim9* (Figure 8G1 and 8G2). In this group, the most subtle changes are shown by *Sst* (Figures 8B1 and 8B2). In wild type, neocortical neurons expressing Sst are localized to the marginal zone (MZ) and the subplate (SPL, open arrows in Figure 8B1) as well as to the IZ (black arrows in Figure 8B1). In the mutant, however, Sst-expressing neurons can be found only in the MZ (open arrow in Figure 8B2). Transcription factor Arx is expressed in scattered neurons at every level of the developing wild-type cortex, and particularly in the MZ (Figure 8D1). qPCR showed an overall increase in signal in the mutant neocortex (Figure S1), and ISH indicated that this increase does not occur in the scattered Arx-expressing cells of the IZ and MZ, but takes the form of a novel Arx-expressing domain presumably coinciding with the SVZ of the mutant cortex (open arrow in Figure 8D2). It is possible that this novel band of Arx-expressing neurons contains basal ganglia neurons that in the Pax6^sey/sey^ cortex could have migrated through the pallio-subpallial barrier and invaded the cortex [21]. In the cases of *Neurod1*, *Neurod6*, and *Trim9* (Figure 8E–8G) the mutant cortex did not show novel expression domains but rather a loss of very specific expression regions (open arrows in Figure 8E1-8G1). *Neurog1*, *Neurog2*, and *Pde1b* represent genes with relatively simple spatial expression patterns in the wild-type neocortex (Figure 8I1-8K1). Accordingly, changes in pattern seen in Pax6^sey/sey^ are simple, taking the form of a disappearance of the single expression domain (Figure 8I2-8K2). Finally, *D930015E06Rik*, *Lrp5*, and *Fzd2* are genes showing quite widespread expression in wild-type neocortex (Figure 8L1-8N1). ISH indicates a global reduction in expression intensity in Pax6^sey/sey^ cortex (Figure 8L2-8N2). Of note, even in these cases, ISH suggests a relative upregulation of expression in the subplate of Pax6^sey/sey^ cortex (open arrows in Figure 8M2 and 8N2). In summary, the expression of half of the 30 candidate Pax6-regulated genes is changed in the cortex of Pax6^sey/sey^, indicating that a combination of robotic ISH and hierarchical clustering of annotations can be used for prioritizing candidate genes for a next round of analysis.

Next, we investigated whether cluster 7 showed an enrichment of Pax6-regulated genes relative to other clusters. Cluster 10 contains 215 genes, many of which are expressed in the neocortex (Figure 4). A sample of 41 genes (Table S4) was selected that colocalized with Pax6 in the E14.5 neocortex in a manner similar to that described for the 30 genes of cluster 7 (Figure 7). Applying the same criteria as were used for cluster 7, we find that only ten (24%) of the 41 cluster 10 genes exhibit an altered expression pattern in the E15.5 neocortex of Pax6^sey/sey^ mice (bold marked genes in Table S4). Compared to 16 of 30 differentially expressed genes (53%) in cluster 7, hierarchical clustering resulted in significant enrichment of putative Pax6 targets among cortically expressed genes in cluster 7 (*p* < 0.02,Fisher's exact test, one-tailed).

### Pax6 Binding Site Identification

Differential gene expression in Pax6^sey/sey^ cortex (Figure 8) raised the possibility that the genes in question harbor Pax6 binding sites. In the absence of Pax6, these binding sites would no longer be occupied, and this could directly affect gene expression. Alternatively, some of the 16 genes could be downstream of a Pax6-regulated gene thus implying an indirect role of Pax6. We searched for sites that are conserved between human and mouse [8] and subsequently examined whether the predicted sites were capable of binding to a fusion protein composed of a Pax6-paired domain (PD) and a glutathione-S-transferase (GST) tag (Pax6-PD-GST). The DNA binding affinities of either the full-length Pax6 protein or of an Escherichia coli made Pax6-PD-GST are similar [22], prompting us to use the fusion protein for binding site analysis. While such DNA binding experiments would not prove functionality of Pax6 binding in the embryo, they provide a rational procedure to identify genes that share a regulatory element and, at least in part, validate the selection approach described in the previous sections.

**Figure 9:**
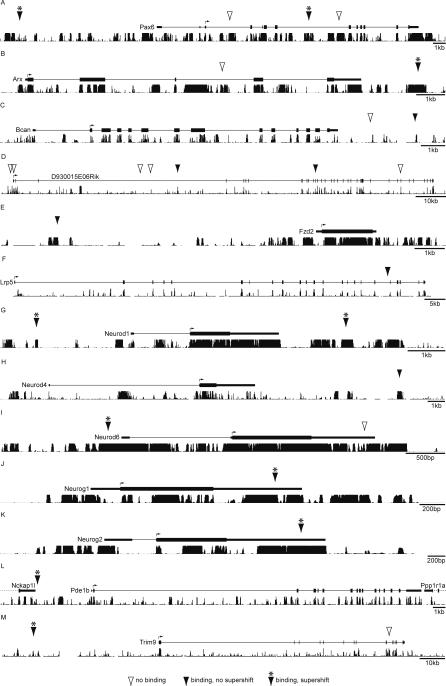
Location of Conserved Pax6-Paired Domain Binding Sites Arrowheads indicate the location of human/mouse conserved Pax6 binding sites relative to exons of the corresponding genes. Conservation profiles indicate sequence conservation and were generated using the UCSC genome browser [23,24]. Arrow heads indicate conserved Pax6-paired domain binding sites that were examined by EMSA. Black arrowheads and asterisks indicate band shifting and supershifting, black arrowheads indicate band shifting only, white arrowheads indicate absence of bandshifting.

**Figure 10:**
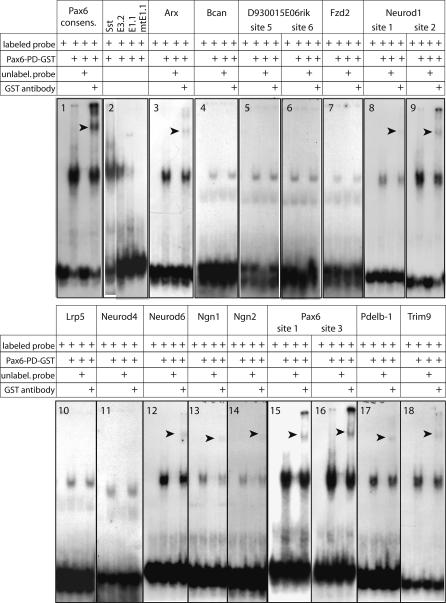
EMSA Results for Pax6 on Our Candidate Genes Autoradiograms with results of EMSA experiments for genes containing a conserved Pax6-paired domain site. Each experiment—except for panel 2—has four lanes, which are (from left to right): (1) probe only; (2) probe incubated together with Pax6-PD-GST protein; (3) probe incubated with Pax6-PD-GST protein first in the presence of 50-fold excess of unlabeled fusion probe, and then with labeled probe; (4) probe incubated with protein plus anti-GST antibody; the supershifted band is indicated by an arrowhead. In case of multiple binding sites in a single gene, the left-most autoradiograph corresponds to the most 5' binding site.

**Figure 11:**
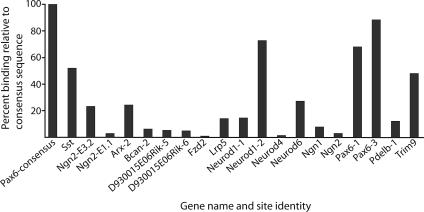
Estimated Binding Affinity Based on EMSA Results Estimates of binding affinity of Pax6-PD-GST to the binding sites found in each of the candidate genes relative to the consensus sequence. Ordinate: percent binding relative to consensus sequence; abscissa: gene name and site identity.

The extent of sequence conservation between human/mouse in the loci of the 16 differentially expressed genes was determined using the University of California Santa Cruz (UCSC) genome browser [23,24] (Figure 9). Human and mouse genomic sequences delineated by this browser were first aligned using the zPicture tool of the Dcode.org Comparative Genomics Center (http://zpicture.dcode.org) [25] followed by a definition of Pax6 binding sites in the conserved regions with the help of TRANSFAC professional (V10.2) [26]. Previous work had already demonstrated Pax6 binding sites in *Sst* [27] and *Neurog2* [28]. Overall, we identified in 14 of the 16 differentially expressed genes a total of 27 Pax6-conserved binding sites (Figure 9 open and filled triangles; see Table S3 for mouse-predicted binding site–DNA sequences). All predicted sites were examined by electromobility shift assays (EMSAs). Before examining the 27 sites, we carried out a series of validation experiments using previously characterized Pax6-paired domain binding sites. These sites were ACATTCACGCATGACTGACT derived from the Pax6-binding consensus sequence (ANNTTCACGCWTSANTKMNY) [22], a Pax6 binding site identified in the *Sst* gene (Table S3) [27], and two sites in the *Neurog2* gene (termed E3.2 and E1.1 [28]; Table S3). As expected, the consensus sequence-derived positive control yielded a very robust signal. The fragment was markedly shifted, could be competed with a 50-fold excess of nonlabeled DNA, and was supershifted using an anti-GST antibody (Figure 10, panel 1). Next, we analyzed the Pax6 binding sites in the *Sst* and *Neurog2* genes. The *Sst* site was shifted by Pax6-PD-GST and so were E1.1 and E3.2 (Figure 10, panel 2). Of note, E1.1 had previously been functionally validated by transgenesis in vivo [27]. Relative to the consensus sequence, the amount of shifted complex, albeit significant, was low. Mutated E1.1 was not able to bind to Pax6-PD-GST fusion protein (Figure 10, panel 2, last lane). Quantification of the shifted band showed that relative to the positive control 2.8 and 23% of the E1.1 and E3.2 oligonucleotides were protein bound (Figure 11).

All 27 Pax6 binding sites predicted by bioinformatics were subjected to the same analysis as described for the controls. For 11 sites, we could not detect any binding under our conditions (open triangles in Figure 8, for DNA sequences see Table S3). For the remaining 16 sites, we observed binding to a variable degree (Figures 10 and 11). For example, in the case of Arx, the percent of radioactive site bound was 24% relative to the consensus sequence (Figure 11). This band could be competed away with 50-fold excess of nonlabeled binding site DNA and be supershifted, albeit weakly, with an anti-GST antibody (Figure 10, panel 3). Quantitatively similar shifts were observed with *Neurod1* (site 2), *Neurod6*, *Pde1b* (site 1), and *Trim9*. Weak binding was seen for *Bcan*, *D930015E06Rik*, *Fzd2*, and *Neurod4*. Taken together, 12 new genes harboring Pax6-PD binding sequences were identified, in addition to those previously described in *Neurog2* and *Sst*. Thus, of the 80 genes that hierarchical clustering grouped with Pax6, 14 have experimentally verified Pax6-PD binding sites.

## Discussion

Biological processes are driven by networks of genes and proteins. This view has emerged from diverse observations including biochemical pathway analysis, genetic and protein interaction screens, and microarray hybridization (see [29] for a review). Recently, several large scale ISH analyses have been undertaken (e.g., [3,30]), which will contribute to deepening our understanding of biological networks and, in addition, offer strategies to discover and validate networks. To capture the complexity of ISH-generated expression patterns, it is paramount to catalogue expression data in a standardized format (e.g., an atlas) in which location and strength of expression for each gene are annotated using either a controlled vocabulary (this study and [30]) or a structure-based automated annotation [3,31]. The present study demonstrates that an annotated atlas of ISH expression patterns, when appropriately mined and complemented with expression studies in mutant tissue and biochemical assays, offers a novel strategy to identify components of a Pax6-controlled gene regulatory network.

### Assessment of Approach

Our annotated and searchable spatial gene expression atlas of the midgestation mouse embryo is a useful resource allowing scientists to search and view expression patterns of individual genes. However, an annotated atlas also provides an entryway to questions that reach far beyond merely viewing expression patterns of individual genes. We demonstrated that hierarchical clustering of the annotation of expression patterns can lead to dendrograms grouping tissues and genes (Figures 4–6). In the case of organs, the outcome of clustering is striking in that tissues of common origin and function cosegregate, as is the case for the various constituents of the central nervous system. Such convergence not only reflects the fact that central nervous system tissues are chiefly composed of neurons that share a characteristic physiology, but that all neurons derive from neuroectoderm (for a discussion of this issue, see also [5]). Subregions of the developing brain known to be closely related by lineage and function (e.g., hippocampus, neocortex, and olfactory bulb) form neighboring branches of the dendrogram of organs. Tissues that owe their architecture and function to mesenchymal epithelial interactions (e.g., lung, kidney, salivary gland, teeth, and whisker follicles) tend to be neighbors in the organ dendrogram. Hence clustering of annotations of patterns recreates an authentic body plan of the organism, a result reminiscent of that reported for *Drosophila* [30].

Because clustering is successful in recapitulation of the body plan, it is tempting to assume it also assembles genes in meaningful groups. To test this possibility we asked whether genes bunched in one of the 12 groups (Figure 4) belong to a regulatory cascade. We selected group 7, one of the larger and hence representative groups containing genes predominantly expressed in the central nervous system. A total of 23 group 7 genes encode transcription factors, including Pax6, which is one of the most strongly conserved master mediators of eye and brain development in metazoans (see Introduction). Clustering encompassed all annotated organs, but because regulatory networks are likely to exhibit some level of organ specificity, we focused on genes coexpressed with *Pax6* during the development of the cortex, the seat of higher cognitive abilities. We found that 30 group 7 genes were coexpressed with *Pax6*, 14 of which not only showed altered cortical expression in *Pax6^sey/sey^*embryos, but also had experimentally verifiable Pax6-paired domain binding sites.

Holm et al. [32] have used microarrays to identify genes that are differentially expressed in the telencephalon of wild-type and *Pax6^sey/sey^*embryos at E15. These data can be compared with our ISH analyses of cortical changes in gene expression at E15.5 (Figure 8). Among the ∼100 transcripts identified by [32], three of them—*Neurog1*, *Neurog2*, and *Arx*—are also in our list. qPCR analysis suggests that *Neurog1* and *Neurog2* are downregulated in *Pax6^sey/sey^* embryos by more than an order of magnitude (Figure S1). By contrast, these data indicate that *Arx* is upregulated in the mutant cortex by a factor of 4. Microarray results show qualitatively similar results, although the change in expression is less pronounced [32]. The same authors [32] found differential regulation of several genes that are contained in the 1K dataset, but did not cluster with *Pax6*. Examples are *Sema5a* and *Cxcl12*, which are expressed in the VZ of the neocortex and are downregulated in *Pax6^sey/sey^* embryos (M. Warnecke, J. Oldekamp, G. Alvarez-Bolado, and G. Eichele, unpublished data). Both, *Sema5a* and *Cxcl12* reside in cluster 10, a cluster that includes genes expressed in nervous system and in mesoderm-derived structures (Figure 4). One way to avoid escaping of such genes is to restrict clustering to a subset of tissue types.

Our strategy for discovering components of networks is not restricted to transcription factors; e.g., kinases and their substrates could be identified by hierarchical clustering, which could be followed up by biochemistry using kinase assays. In this particular case, expression data could be combined with information from the protein interactome that identifies enzyme-substrate interactions. A prerequisite for a successful application of our strategy is a significant degree of regional expression of regulators and their targets.

### Initial Biological Assessment of Putative Pax6 Targets

Because of the severity of its cortical, pancreatic, and ocular phenotypes, the *Pax6^sey/sey^* mutant has become emblematic for the molecular genetic approach to development. The cellular processes in which Pax6 has a key regulatory role include cell proliferation, adhesion, and migration [33]. Pax6 targets relevant to eye and pancreas development have been identified [11,33,34], but in the cortex the Pax6 network has proved most difficult to unravel; *Neurog2* is the only known direct target of Pax6 in this tissue [28]. Although the expression of adhesion-related proteins Cdh4 (R-Cadherin) and Fut9 is dramatically decreased in the *Pax6^sey/sey^* cortex [35–37], it is not known whether these genes are direct Pax6 targets. Among the 12 new cortically expressed genes containing a Pax6-paired domain binding site, six encode transcription factor proteins (Arx, Neurod1, Neurod4, Neurod6, Neurog1, and Neurog2). This advocates for a multilayered activation cascade, i.e., a network of considerable complexity. Except for Arx, a homeodomain transcription factor, the other transcription factors uncovered by this study belong to the basic helix-loop-helix (bHLH) family. These genes, also termed proneural genes, are essential regulators of neurogenesis, coordinating the acquisition of the neuronal fate—the specific neuronal subtype identity appropriate for birth date and location of a neuron [38].

Arx is required for proper forebrain development in humans [39] and mouse [40]. Expression of Arx characterizes a group of neurons that migrate tangentially from the basal ganglia into the cortex. Thus, augmented Arx expression in the IZ of *Pax6^sey/sey^* cortex (Figure 8D1 and 8D2) could originate from increased migration of Arx-positive cells. Because Arx is also expressed in cells resident in the cortex, the Arx upregulation observed in the *Pax6^sey/sey^* cortex could be caused by a direct de-repression of *Arx*, a scenario supported by our finding that Pax6 binds to *Arx* regulatory regions. Previous studies have indicated that *Arx* positively regulates cell proliferation in the VZ [40] and the upregulation of this gene in the cortex of *Pax6^sey/sey^* may account for the thickening of the germinative zone in this mutant [41].

*Pde1b* is a calcium- and calmodulin-dependent phosphodiesterase and inhibits cyclic nucleotide signaling. Although its role in the neuroepithelium is unknown, this gene could represent a class of effectors in the network by which Pax6, through binding to the *Pde1b* promoter, would regulate signaling. Wnt proteins are components of potent signaling cascades with major roles in the development of the brain [42–46]. Genes in the Wnt pathway (*Wnt7b* and *Tcf7l2-Tcf4*) have been implicated in Pax6 function in the diencephalon [47,48]. Cluster 7 contains both of these genes and additionally *Wnt1*, *Wnt2b*, *Wnt3*, *Wnt3a*, *Wnt7a*, *Fzd2*, and *Lrp5. Fzd2* and *Lrp5* are Wnt coreceptors that are coexpressed with *Pax6* in the cortex, and the corresponding genes have Pax6-paired domain binding sites. The *Wnt* pathway is an essential regulator of telencephalic “dorsalization,” a process that confers cortex-forming capabilities to the dorsal half of the anterior neural tube [49,50]. Our study would thus place the *Pax6* cascade at the intersection with the *Wnt* pathway. It is rational for a pathway regulating the subdivision of the cortical neuroepithelium into neocortex and hippocampus to intersect with a pathway involved in activation of proneural genes (*Neurod* and *Neurog* families) that confer region-specific neuronal subtype traits.

*Bcan* (*Brevican* or *Cspg7*) codes for a brain-specific chondroitin sulphate proteoglycan abundant in the extracellular matrix and having a function in the development of axonal tracts [51,52]. Intriguingly, the expression pattern of two other chondroitin sulphate proteoglycans (*Cspg3-neurocan* and *Ptprz1-phosphacan*) are altered in the *Emx2* mutant [53], which is another major transcription factor mutant with a cortical phenotype. *Emx2* is thought to work in balance with *Pax6* to partition the cortex into specialized functional areas [54]. These data suggest that extracellular matrix proteoglycans are important effectors in the networks responsible for the specification of cortical subdivisions and underline the central role of adhesion events in this process [33].

*Trim9* encodes a brain-specific member of the “tripartite motif” protein family, which binds to the cytoskeletal microtubules [55,56] and could possibly represent an effector molecule by which Pax6 regulates cytoskeletal function during cell migration or polarization. *Trim9* expression marks two sharply delimited bands in the developing cortex, the cortical plate and the SVZ (Figure 8G1). The latter disappears in the *Pax6* mutant (Figure 8G2) indicating a lack of proper differentiation in the SVZ, or perhaps the complete absence of this essential germinal compartment, consistent with previous observations [57]. The expression patterns of genes such as *Eomes* and *Igfbnl1* are affected in the cortex of *Pax6^sey/sey^*embryos but no Pax6-paired domain binding sites were found within a conserved region.

The present study identifies several genes expressed in the developing cortex that have Pax6-paired domain binding sites. DNA binding as assessed by EMSA shows that binding affinities to the various DNA fragments derived from human/mouse sequence conservation varies considerably. It can readily be seen from Figures 10 and 11 that strength of DNA binding of the Pax6-paired domain to particular DNA sequences is often only a fraction of that of the consensus sequence. It should be noted, however, that the in vivo validated site E1.1 of the *Neurog2* gene [28] showed binding similar to that seen in e.g., *Neurog*1. Additional experiments using transgenic mice will be required to establish functional relevance of the candidate binding sites identified in the present study.

### Conclusions

Experimental and computational methods employed in the present work provide a partial list of components of the cortical Pax6 network. A next logical step will be to apply the approach used in the present study to mice mutated in the putative Pax6 target genes identified here. This will eventually provide a portrait of the undoubtedly very complex Pax6 regulatory network. The question is raised of how many components could the Pax6 network be composed? The present study is based on expression patterns of approximately 8% of genes expressed in the midgestation mouse embryo and has led to 14 genes regulated by Pax6 in the developing cortex. Hence, upon completion of the E14.5 transcriptome atlas, the number of Pax6-regulated genes may reach a figure of ∼200, not including those controlled by transcription factors downstream of Pax6.

## Materials and Methods

### Automated ISH.

For automated ISH, mouse embryos were embedded in Tissue-Tek O.C.T. (Sakura), quick frozen, and sectioned at 25-μm thickness. Hybridization was performed on a Tecan ISH robot. Nonradioactive, digoxigenin-tagged riboprobes were detected by a two-step chromogenic catalyzed reporter deposition protocol. Riboprobes were generated by standard methods and were usually 700–1,000 nucleotides long [18]. Template sequences are available at www.genepaint.org [20].

### Mouse strains.

E14.5 mouse embryos belonged to either the NMRI or C57/BL6 strains. The strain for each expression pattern is given in genepaint.org and can be found in the info box on the set viewer page of the gene in question. The E15.5 wild-type and *Pax6^sey/sey^* embryos were littermates. Small eye is a spontaneous mutation [58] kept in the genetic background C57BL/6J × DBA/2J.

### Gene ontology analysis.

Datasets were analyzed with the DAVID GoChart tool [59]. Molecular functions in Figure 2A correspond to GoChart level 2.

### qPCR and microarrays.

For real-time (RT)-qPCR, cortex tissue was dissected from E15.5 *Pax6^sey/sey^* embryos and wild-type littermates. Total RNA was prepared from pooled wild-type or *Pax6^sey/sey^* tissue samples. Reverse transcription was accomplished via standard methods. Real-time PCR was carried out with an iCycler (BioRad) using a SYBR-green quantification protocol [60]. Primers specific for candidate genes were used to determine expression levels in wild type and *Pax6^sey/sey^*. Expression levels were normalized to the house-keeping gene *EF1*α. Error bars in Figure S1 are standard deviations of replicates. Expression differences were in all cases statistically significant (*p* < 0.001) as determined by Student's t-test. Microarray analysis was performed with RNA extracted from E14.5 whole embryos and Mouse Genome 430 2.0 microarrays (Affymetrix) according to standard methods. Analysis was completed in duplicate, and genes were considered expressed if both replicates had a “present” absolute call (detection value *p* < 0.05).

### Identification and validation of Pax-paired domain binding sites.

Putative binding sites in human-mouse conserved regions of the candidate genes were identified with the help of the TRANSFAC (binding sites) and UCSC Genome Browser [23,24] (conserved regions) databases.

### Cluster analysis.

Dendrograms for genes and anatomical regions were produced with R Project for Statistical Computing software (http://www.r-project.org/) using Ward's clustering [61]. Distances between genes and between anatomical terms were calculated using Python programming language (http://www.python.org/). The distance metric between each pair of genes was defined on the basis of the strength and pattern of expression at each anatomical region. In a similar fashion, the distance between each anatomical region was defined on the basis of the strength and pattern of expression for every gene in that region (see Tables S1–S3 in Supplementary Material for details).

### EMSA.

EMSA reactions were carried out according to [22]. The 2.48-kb *Pax6* cDNA was obtained from Luc St.Onge and Peter Gruss (Max Planck Institute of Biophysical Chemistry, Göttingen, Germany). A 562-bp (nts 135–697) paired domain-containing BamHI-XmaI fragment was cloned in frame with the GST tag into the “pGEM 5×1” expression vector (Promega). The Pax6-PD-GST fusion protein was expressed in BL21 bacteria and extracted according to standard protocols. It was found that 1 μl of bacterial lysate contained a total of 9.4 μg of total protein (Bradford method, using bovine serum as standard). To every binding site oligonucleotide (Table S3) two additional sequences were added: on the 5'end, CGC was added as a spacer, and to the 3'end, the 20-mer GGA TCA AGA GCT ACC AAC TC was added allowing primer extension with Klenow DNA polymerase. Radiolabeling with α^32^P-dATP was carried out with the Klenow DNA polymerase using 10 pmol of binding site oligonucleotide and oligonucleotide primer complementary to the 20-mer added to the 3' end. Labeled DNA was purified with G-50 columns. One microliter of labeled DNA (about 10^5^ cpm) was incubated on ice for 30 min with 1 μl of bacterial lysate containing Pax6-PD-GST. For the competition assay, 50-fold excess of unlabeled DNA was incubated with 1 μl of bacterial lysate first, then 1 μl of DNA was added, and the mixture was incubated on ice for another 30 min. For the supershift assay the labeled binding site was incubated with bacterial lysate containing Pax6-PD-GST fusion protein on ice for 30 min, then anti-GST antibody was added and incubated for another 30 min. Finally, 5 μl of each product were loaded on a precooled 5% polyacrylamide gel and electrophoresed at 4 °C and 150 V for 2.5 h.

### Quantification of bound and unbound DNA binding sites.

Autoradiographs and gels were aligned, shifted bands and free probe bands were cut out, radioactivity contained therein was measured in a scintillation counter, and the ratio of shifted to total radioactivity was calculated for each experiment. This ratio was normalized to that obtained from binding of Pax6-PD-GST to the Pax6 consensus sequence (“positive control” in Table S3), which was included in all experiments. Also included in each analysis was a negative control, binding of Pax6-PD-GST to mt-E1.1 of *Neurog2* (Table S3).

## Supporting Information

Figure S1 Differential Gene Expression in the Cortex Quantified by Real-Time qPCR Differential expression of eight of the16 genes shown in Figure 8 was validated by qPCR.
(253 KB PDF)

Table S1 1K ISH Dataset (1.2 MB DOC)

Table S2 Filtering of 1K ISH Dataset for Candidate Pax6 Target Genes (127 KB DOC)

Table S3 DNA Sequence of the Experimentally Tested Pax6 Binding Sites in the Selected Target Genes (87 KB DOC)

Table S4 Cluster 10 Genes Coexpressed with Pax6 in the Neocortex at E14.5 Genes marked in bold are differentially expressed in *Pax6^sey/sey^*.
(31 KB DOC)

Text S1 Hierarchical Clustering (59 KB DOC)

###  Accession Numbers

The accession number mentioned in this paper from the National Center for Biotechnology Information Gene Expression Omnibus (NCBI GEO) (http://www.ncbi.nlm.nih.gov/geo) is GSE6081.

## Abbreviations

EMSA: electromobility shift assay
GST: glutathione-S-transferase
ISH: in situ hybridization
IZ: intermediate zone
MZ: marginal zone
PD: paired domain
qPCR: quantitative PCR
SVZ: subventricular zone
VZ: ventricular zone
