# Supplementary material for: Regulatory Pathway Analysis by High-Throughput In Situ Hybridization
Source: PLoS Genet. 2007 Oct 19;3(10):e178. doi: 10.1371/journal.pgen.0030178 (PMC2041993; doi:10.1371/journal.pgen.0030178)
Supplement: Supplementary file 1 [file pgen.0030178.sd001.doc]

Supplementary Material Hierarchical Clustering

**Review of annotation scheme.**

We used a standardized annotation scheme to describe the expression pattern of each gene (Visel et al., 2004). Briefly, for each gene and each anatomical region, the gene expression patterns and strengths were visually annotated. Patterns are characterized as Regional (R), Scattered (S), or Ubiquitous (U). The strengths, based on the degree to which cells were filled with the dye signal, are Strong (+++), Moderate (++), or Weak (+). If no gene expression is visible, the region for that gene is be annotated as Not Detected (-).

**Distance metric calculation method between two genes.**

The distance metric, *D*, between any two genes is based on their expression pattern across all leaf anatomical regions. The distance metric is calculated from the similarity score, *S*, between the two genes:

*D =* (20 / (*S* + 0.1) ) - 18

Where *D* is rounded to the nearest integer and the values of the constants in the above equation are designed for *D* to be within the range of 0 to 182 when the similarity score is between 1 and 0. Distance was capped at a finite value as the inclusion of an infinite distance value could prevent proper clustering.

The similarity score between any two genes is calculated as the sum of local pattern similarity values, m, and the potential total pattern similarity values, t:

*S* = ∑ m / ∑ t

Under this method, the maximum value for *S* is 1, and the minimum value for *S* is 0. The calculation of the values of m and t are now described in detail.

The method for calculating distance between the gene expression patterns of two genes across all anatomical regions was designed to meet the following criteria:

1. Genes that do not express in any of the same anatomical regions are maximally distant.
2. The distance metric is adaptive to the range of expression strengths for a gene.
3. Pattern is secondary to strength

To address criterion 1, m and t are both set to zero for anatomical regions that have no expression in either of the pair of genes being considered. In this way, anatomical regions not expressing either gene play no role in the calculation of similarity or distance. While one may argue that the shared lack of expression in a region is a form of similarity, this criterion is necessary to prevent two genes that express in very few non-overlapping locations from being considered at all similar.

The rationale for criterion 2 is that in some cases a gene is observed to express only in a weak fashion. Because a gene’s expression strength is most significant when compared to the same gene, from a self-relative perspective a gene that only expresses weakly is virtually identical in expression pattern to another gene that only expresses strongly, and only across the same locations that the first gene expresses. For this reason, special scoring tables for m and t are created to handle genes that only express weakly and raise them in significance to equal genes that express strongly.

In most cases, t, which serves as a weighting factor for the local comparison, is set to 7. However, as an additional measure to satisfy criterion 2, in some instances t is set to lower values to decrease the impact of that location on the similarity score. E.g. for two genes with locations of strong expression, locations where both have weak expression are weighted less.

The last criterion leads to pattern (R, U, S) augmenting the similarity score to a lesser extent than the expression signal strength. This is due to the reasonable likelihood that there are cellular overlaps in expression regardless of the pattern. Ubiquitous patterns were rarely annotated and were combined into the same category (R/U) for the purpose of the calculations.

The values of m and t are listed in the following three tables. The choice of table to use in the calculation depend upon the maximum strength of gene expression in the two genes as per criterion 2.

Supplementary Table I. If both genes did not moderately or strongly express in any location, then at each location the following values of m and t would be used to calculate the similarity between two genes.

| Gene1/Gene2 | - | S+ | R/U+ |
| --- | --- | --- | --- |
| - | m=0  t=0 | m=5  t=7 | m=5  t=7 |
| S+ | m=5  t=7 | m=7  t=7 | m=0  t=7 |
| R/U+ | m=5  t=7 | m=0  t=7 | m=7  t=7 |

Supplementary Table II. If exactly one of the two genes did not moderately or strongly express in any location, then at each location the following values of m and t would be used to calculate the similarity between two genes.

| Gene1/Gene2 | - | S+ | R/U+ |
| --- | --- | --- | --- |
| - | m=0  t=0 | m=0  t=0 | m=0  t=0 |
| S+ | m=0  t=0 | m=2  t=4 | m=1  t=4 |
| S++ | m=0  t=7 | m=7  t=7 | m=5  t=7 |
| S+++ | m=0  t=7 | m=7  t=7 | m=5  t=7 |
| R/U+ | m=0  t=0 | m=1  t=4 | m=2  t=4 |
| R/U++ | m=0  t=7 | m=5  t=7 | m=7  t=7 |
| R/U+++ | m=0  t=7 | m=5  t=7 | m=7  t=7 |

Supplementary Table III. If both genes express moderately or strongly in at least one location, then at each location the following values of m and t would be used to calculate the similarity between two genes.

| Gene1/Gene2 | - | S+ | S++ | S+++ | R/U+ | R/U++ | R/U+++ |
| --- | --- | --- | --- | --- | --- | --- | --- |
| - | m=0  t=0 | m=0  t=0 | m=0  t=7 | m=0  t=7 | m=0  t=0 | m=0  t=7 | m=0  t=7 |
| S+ | m=0  t=0 | m=2  t=2 | m=1  t=7 | m=0  t=7 | m=1  t=2 | m=1  t=7 | m=0  t=7 |
| S++ | m=0  t=7 | m=1  t=7 | m=7  t=7 | m=6  t=7 | m=1  t=7 | m=5  t=7 | m=4  t=7 |
| S+++ | m=0  t=7 | m=0  t=7 | m=6  t=7 | m=7  t=7 | m=0  t=7 | m=4  t=7 | m=5  t=7 |
| R/U+ | m=0  t=0 | m=1  t=2 | m=1  t=7 | m=0  t=7 | m=2  t=2 | m=1  t=7 | m=0  t=7 |
| R/U++ | m=0  t=7 | m=1  t=7 | m=5  t=7 | m=4  t=7 | m=1  t=7 | m=7  t=7 | m=6  t=7 |
| R/U+++ | m=0  t=7 | m=0  t=7 | m=4  t=7 | m=5  t=7 | m=0  t=7 | m=6  t=7 | m=7  t=7 |

**Distance metric calculation method between two anatomical regions.**

The same approach, equations, and tables were utilized for calculating the distance metric between two anatomical regions with the following modification: instead of locations, the values and table criteria were based upon the expression patterns across all genes for any two anatomical regions. Simply put, switch “location” with “gene”.
