# Supplementary material for: Regulatory Pathway Analysis by High-Throughput In Situ Hybridization
Source: PLoS Genet. 2007 Oct 19;3(10):e178. doi: 10.1371/journal.pgen.0030178 (PMC2041993; doi:10.1371/journal.pgen.0030178)
Supplement: Supplementary file 2 [file pgen.0030178.st001.doc]

| Supplemental Table 1 1K ISH Data Set | | | |
| --- | --- | --- | --- |
| **GenePaint Set** | **Gene Symbol** | **Gene Name** | **GeneID**  **(Entrez)** |
| HD37 | 0610009I22Rik | RIKEN cDNA 0610009I22 gene | 66586 |
| MY248 | 0610016J10Rik | RIKEN cDNA 0610016J10 gene | 76890 |
| MH702 | 1110003F05Rik | RIKEN cDNA 1110003F05 gene | 66145 |
| MH382 | 1110030H18Rik | RIKEN cDNA 1110030H18 gene | 68666 |
| MH903 | 1110031M08Rik | RIKEN cDNA 1110031M08 gene | 68693 |
| HD58 | 1110036O03Rik | RIKEN cDNA 1110036O03 gene | 66180 |
| MY190 | 1190017O12Rik | RIKEN cDNA 1190017O12 gene | 68936 |
| MH1690 | 1200008O12Rik | RIKEN cDNA 1200008O12 gene | 74107 |
| MH1198 | 1200016E24Rik | RIKEN cDNA 1200016E24 gene | 319202 |
| MH457 | 1300007L22Rik | RIKEN cDNA 1300007L22 gene | 71774 |
| MY193 | 1500032D16Rik | RIKEN cDNA 1500032D16 gene | 78330 |
| MH685 | 1700013G20Rik | RIKEN cDNA 1700013G20 gene | 75516 |
| MH705 | 1700086L19Rik | RIKEN cDNA 1700086L19 gene | 74284 |
| MH919 | 1700111I05Rik | RIKEN cDNA 1700111I05 gene | 76629 |
| MY277 | 1810007M14Rik | RIKEN cDNA 1810007M14 gene | 67367 |
| MY191 | 1810008A18Rik | RIKEN cDNA 1810008A18 gene | 108707 |
| ST73 | 1810009A16Rik | RIKEN cDNA 1810009A16 gene | 69116 |
| MH825 | 1810009M01Rik | RIKEN cDNA 1810009M01 gene | 65963 |
| ST29 | 1810020G14Rik | RIKEN cDNA 1810020G14 gene | 66272 |
| MY309 | 1810043G02Rik | RIKEN cDNA 1810043G02 gene | 67884 |
| MH1199 | 1810073G14Rik | RIKEN cDNA 1810073G14 gene | 67726 |
| MH508 | 1810074P20Rik | RIKEN cDNA 1810074P20 gene | 67490 |
| MH976 | 2010012C16Rik | RIKEN cDNA 2010012C16 gene | 66441 |
| MH998 | 2010109N14Rik | RIKEN cDNA 2010109N14 gene | 69895 |
| MH596 | 2410003C20Rik | RIKEN cDNA 2410003C20 gene | 66983 |
| MH996 | 2410004I17Rik | RIKEN cDNA 2410004I17 gene | 71988 |
| HD50 | 2410146L05Rik | RIKEN cDNA 2410146L05 gene | 67968 |
| MY14 | 2600005C20Rik | RIKEN cDNA 2600005C20 gene | 72462 |
| MH171 | 2610002F03Rik | RIKEN cDNA 2610002F03 gene | 72091 |
| MH561 | 2610034M16Rik | RIKEN cDNA 2610034M16 gene | 69239 |
| MY262 | 2610039C10Rik | RIKEN cDNA 2610039C10 gene | 66578 |
| MY131 | 2610510B01Rik | RIKEN cDNA 2610510B01 gene | 70028 |
| MH1262 | 2700038I16Rik | RIKEN cDNA 2700038I16 gene | 72611 |
| MH706 | 2810418N01Rik | RIKEN cDNA 2810418N01 gene | 69270 |
| MH707 | 2810426N06Rik | RIKEN cDNA 2810426N06 gene | 67607 |
| MH1154 | 2810449C10Rik | RIKEN cDNA 2810449C10 gene | 72758 |
| MH687 | 2810474O19Rik | RIKEN cDNA 2810474O19 gene | 67246 |
| HD34 | 2900042E01Rik | RIKEN cDNA 2900042E01 gene | 72927 |
| MH1000 | 2900045N06Rik | RIKEN cDNA 2900045N06 gene | 72895 |
| MH898 | 2900073H19Rik | RIKEN cDNA 2900073H19 gene | 68205 |
| MH708 | 3110003A17Rik | RIKEN cDNA 3110003A17 gene | 73112 |
| ST80 | 4632413K17Rik | RIKEN cDNA 4632413K17 gene | 216440 |
| ST87 | 4732437J24Rik | RIKEN cDNA 4732437J24 gene | 214063 |
| MY278 | 4921511H13Rik | RIKEN cDNA 4921511H13 gene | 207932 |
| MH562 | 4930422G04Rik | RIKEN cDNA 4930422G04 gene | 71643 |
| HD74 | 4930431B11Rik | RIKEN cDNA 4930431B11 gene | 75782 |
| MH458 | 4930553M18Rik | RIKEN cDNA 4930553M18 gene | 75316 |
| MY273 | 4931408A02Rik | RIKEN cDNA 4931408A02 gene | 70967 |
| MY298 | 4932438H23Rik | RIKEN cDNA 4932438H23 gene | 74387 |
| MH1188 | 5730590G19Rik | RIKEN cDNA 5730590G19 gene | 77011 |
| MY226 | 5830404H04Rik | RIKEN cDNA A130042E20 gene | 207781 |
| MY243 | 5830445C04Rik | RIKEN cDNA 5830445C04 gene | 67768 |
| MH1186 | 6230416J20Rik | RIKEN cDNA 6230416J20 gene | 230376 |
| MH820 | 6330404F12Rik | RIKEN cDNA 6330404F12 gene | 70758 |
| HD41 | 6330407J23Rik | RIKEN cDNA 6330407J23 gene | 67412 |
| MH1189 | 680402O20Rik | RIKEN cDNA 680402O20 gene | 228829 |
| MH512 | 9030411K21Rik | RIKEN cDNA 9030411K21 gene | 71557 |
| HD14 | 9130024F11Rik | RIKEN cDNA 9130024F11 gene | 78900 |
| MH476 | 9430063L05Rik | RIKEN CDNA 9430063L05 gene | 229622 |
| HD46 | 9930116O05Rik | RIKEN cDNA 9930116O05 gene | 319758 |
| MY310 | A130042E20Rik | RIKEN cDNA A130042E20 gene | 216119 |
| MY310 | A130042E20Rik | RIKEN cDNA A130042E20 gene | 216119 |
| MH227 | A230109K23Rik | RIKEN cDNA A230109K23 gene | 77764 |
| MH738 | A330102H22Rik | RIKEN cDNA A330102H22 gene | 77754 |
| HD29 | Aass | aminoadipate-semialdehyde synthase | 30956 |
| MH675 | Abcc4 | ATP-binding cassette, sub-family C (CFTR/MRP), member 4 | 239273 |
| HD69 | Abcf2 | ATP-binding cassette, sub-family F (GCN20), member 2 | 27407 |
| MY269 | Abcg1 | ATP-binding cassette, sub-family G (WHITE), member 1 | 11307 |
| MH1019 | Acadl | acetyl-Coenzyme A dehydrogenase, long-chain | 11363 |
| MH750 | Acsl1 | acyl-CoA synthetase long-chain family member 1 | 14081 |
| DC37 | Actc1 | actin, alpha, cardiac | 11464 |
| MH1166 | Actn2 | actinin alpha 2 | 11472 |
| MH739 | Actr10 | ARP10 actin-related protein 10 homolog (S. cerevisiae) | 56444 |
| MY280 | Adamts1 | a disintegrin-like and metalloprotease (reprolysin type) with thrombospondin type 1 motif, 1 | 11504 |
| MY69 | Adamts5 | a disintegrin-like and metalloprotease (reprolysin type) with thrombospondin type 1 motif, 5 (aggrecanase-2) | 23794 |
| MH956 | Adcy1 | adenylate cyclase 1 | 216530 |
| MH957 | Adcy2 | adenylate cyclase 2 | 210044 |
| MH849 | Adcy3 | adenylate cyclase 3 | 104111 |
| MH850 | Adcy4 | adenylate cyclase 4 | 104110 |
| MH851 | Adcy5 | adenylate cyclase 5 | 224129 |
| MH852 | Adcy6 | adenylate cyclase 6 | 11512 |
| MH853 | Adcy7 | adenylate cyclase 7 | 11513 |
| MH854 | Adcy8 | adenylate cyclase 8 | 11514 |
| MH855 | Adcy9 | adenylate cyclase 9 | 11515 |
| MH1202 | Agpat3 | 1-acylglycerol-3-phosphate O-acyltransferase 3 | 28169 |
| MH646 | AI606473 | expressed sequence AI606473 | 99686 |
| MY187 | Aire | autoimmune regulator (autoimmune polyendocrinopathy candidiasis ectodermal dystrophy) | 11634 |
| HD47 | Ak4 | adenylate kinase 4 | 11639 |
| MH630 | Akap8 | A kinase (PRKA) anchor protein 8 | 56399 |
| MH1238 | Aldh1a1 | aldehyde dehydrogenase family 1, subfamily A1 | 11668 |
| MH127 | Aldh1a2 | aldehyde dehydrogenase family 1, subfamily A2 | 19378 |
| HD9 | Alg6 | asparagine-linked glycosylation 6 homolog (yeast, alpha-1,3,-glucosyltransferase) | 320438 |
| DA116 | Alx3 | Aristaless 3 | 11694 |
| DA82 | Alx4 | Aristaless 4 | 11695 |
| MH539 | Anp32a | acidic (leucine-rich) nuclear phosphoprotein 32 family, member A | 11737 |
| MH540 | Anxa11 | annexin A11 | 11744 |
| MH711 | Ap2b1 | adaptor-related protein complex 2, beta 1 subunit | 71770 |
| MH475 | Ap2m1 | adaptor protein complex AP-2, mu1 | 11773 |
| MH781 | Apba2 | amyloid beta (A4) precursor protein-binding, family A, member 2 | 11784 |
| MH958 | Apbb1 | amyloid beta (A4) precursor protein-binding, family B, member 1 | 11785 |
| MH605 | Apoa2 | apolipoprotein A-II | 11807 |
| MH1239 | Apoe | apolipoprotein E | 11816 |
| MY188 | App | amyloid beta (A4) precursor protein | 11820 |
| MH1020 | Ar | androgen receptor | 11835 |
| MH712 | Arf3 | ADP-ribosylation factor 3 | 11842 |
| MH737 | Arhgap11a | Rho GTPase activating protein 11A | 228482 |
| MH583 | Arhgap17 | Rho GTPase activating protein 17 | 70497 |
| MH713 | Arhgap5 | Rho GTPase activating protein 5 | 11855 |
| MH460 | Arid1b | AT rich interactive domain 1B (Swi1 like) | 239985 |
| MH88 | Arntl | aryl hydrocarbon receptor nuclear translocator-like | 11865 |
| MH740 | Arx | aristaless related homeobox gene (Drosophila) | 11878 |
| MH714 | Asb4 | ankyrin repeat and SOCS box-containing protein 4 | 65255 |
| DA78 | Ascl2 | achaete-scute complex homolog-like 2 (Drosophila) | 17173 |
| MH606 | Atm | ataxia telangiectasia mutated homolog (human) | 11920 |
| DA128 | Atoh8 | atonal homolog 8 (Drosophila) | 71093 |
| MY293 | Atp5j | ATP synthase, H+ transporting, mitochondrial F0 complex, subunit F | 11957 |
| MH715 | Atp6v0a1 | ATPase, H+ transporting, lysosomal V0 subunit a isoform 1 | 11975 |
| MH710 | AU040576 | expressed sequence AU040576 | 101772 |
| MH641 | Aurka | aurora kinase A | 20878 |
| MH862 | Auts2 | autism susceptibility candidate 2 | 319974 |
| MH417 | Avp | arginine vasopressin | 11998 |
| MH689 | Axin | axin | 12005 |
| MH899 | B130055D15Rik | RIKEN cDNA B130055D15 gene | 78658 |
| MY281 | B230114J08Rik | RIKEN cDNA B230114J08 gene | 77975 |
| MH741 | B930052A04Rik | RIKEN cDNA B930052A04 gene | 320563 |
| MY255 | Bace2 | beta-site APP-cleaving enzyme 2 | 56175 |
| MY256 | Bach1 | BTB and CNC homology 1 | 12013 |
| MH230 | Bambi | BMP and activin membrane-bound inhibitor, homolog (Xenopus laevis) | 68010 |
| MH1006 | BC008103 | cDNA sequence BC008103 | 229524 |
| MH908 | BC026657 | cDNA sequence BC026657 | 208618 |
| MH525 | BC032203 | cDNA sequence BC032203 | 210982 |
| MH701 | BC039093 | cDNA sequence BC039093 | 230761 |
| MH822 | BC059842 | cDNA sequence BC059842 | 230676 |
| MH782 | Bcan | brevican | 12032 |
| MH959 | Bdnf | brain derived neurotrophic factor | 12064 |
| MH631 | Bhlhb5 | basic helix-loop-helix domain containing, class B5 | 59058 |
| MH543 | Birc5 | baculoviral IAP repeat-containing 5 | 11799 |
| MH365 | Bmp4 | bone morphogenetic protein 4 | 12159 |
| MH817 | Bnip3l | BCL2/adenovirus E1B 19kDa-interacting protein 3-like | 12177 |
| MH1097 | Bsg | basigin | 12215 |
| MY185 | Btg3 | B-cell translocation gene 3 | 12228 |
| MH542 | Bub1 | budding uninhibited by benzimidazoles 1 homolog (S. cerevisiae) | 12235 |
| MH823 | Bzrp | benzodiazepine receptor, peripheral | 12257 |
| MH824 | C030019F02Rik | RIKEN cDNA C030019F02 gene | 58237 |
| HD54 | C030033F14Rik | RIKEN cDNA C030033F14 gene | 77386 |
| MH923 | C130060K24Rik | RIKEn cDNA C130060K24 gene | 243407 |
| MH1043 | C130076O07Rik | RIKEN cDNA C130076O07 gene | 319504 |
| MH238 | Calb1 | calbindin-28K | 12307 |
| MH556 | Calb2 | calbindin 2 | 12308 |
| MH742 | Calm1 | calmodulin 1 | 12313 |
| DC46 | Calm2 | calmodulin 2 | 12314 |
| MH778 | Calm3 | calmodulin 3 | 12315 |
| MH440 | Camk2g | calcium/calmodulin -dependent protein kinase II gamma | 12325 |
| MH1098 | Casp9 | caspase 9 | 12371 |
| MH783 | Catna1 | catenin alpha 1 | 12385 |
| MH743 | Catnb | catenin beta | 12387 |
| MH110 | Cbfa2t1h | CBFA2T1 identified gene homolog (human) | 12395 |
| MH863 | Cbfb | core binding factor beta | 12400 |
| MY246 | Cbr1 | carbonyl reductase 1 | 12408 |
| MY205 | Cbr3 | carbonyl reductase 3 | 109857 |
| MY212 | Cbs | cystathionine beta-synthase | 12411 |
| MH604 | Cbx1 | chromobox homolog 1 (Drosophila HP1 beta) | 12412 |
| MH213 | Cbx5 | chromobox homolog 5 (Drosophila HP1a) | 12419 |
| MH784 | Cck | cholecystokinin | 12424 |
| MH545 | Ccna2 | cyclin A2 | 12428 |
| MH416 | Ccnd2 | cyclin D2 | 12444 |
| MH785 | Ccnl2 | cyclin L2 | 56036 |
| MY247 | Cct8 | chaperonin subunit 8 (theta) | 12469 |
| MH673 | Cd1d1 | CD1d1 antigen | 12479 |
| DC31 | Cd81 | CD81 antigen | 12520 |
| MH728 | Cdc14a | CDC14 cell division cycle 14 homolog A (S. cerevisiae) | 229776 |
| MH546 | Cdc2a | cell division cycle 2 homolog A (S. pombe) | 12534 |
| MH729 | Cdca1 | cell division cycle associated 1 | 66977 |
| MH727 | Cdca7 | cell division cycle associated 7 | 66953 |
| DA93 | Cdh1 | Cadherin 1 | 12550 |
| DA84 | Cdh10 | cadherin 10 | 12551 |
| DA85 | Cdh11 | cadherin 11 | 12552 |
| DA94 | Cdh13 | Cadherin 13 | 12554 |
| DA102 | Cdh15 | Cadherin 15 | 12555 |
| DA95 | Cdh2 | cadherin 2 | 12558 |
| DA96 | Cdh20 | Cadherin 20 | 23836 |
| DA100 | Cdh3 | Cadherin 3 | 12560 |
| DA101 | Cdh4 | Cadherin 4 | 12561 |
| DA97 | Cdh5 | cadherin 5 | 12562 |
| DA98 | Cdh6 | Cadherin 6 | 12563 |
| DA115 | Cdh8 | Cadherin 8 | 12564 |
| DA132 | Cdh9 | cadherin 9 | 12565 |
| MH366 | Cdk5 | cyclin-dependent kinase 5 | 12568 |
| MH354 | Cdk5r | cyclin-dependent kinase 5, regulatory subunit (p35) | 12569 |
| DC43 | Cdk5r2 | cyclin-dependent kinase 5, regulatory subunit 2 (p39 | 12570 |
| MH192 | Cdkn1a | cyclin-dependent kinase inhibitor 1A (P21) | 12575 |
| MH510 | Cebpa | CCAAT/enhancer binding protein (C/EBP), alpha | 12606 |
| MH723 | Cenpf | centromere autoantigen F | 108000 |
| MH477 | Centg3 | centaurin, gamma 3 | 213990 |
| MH730 | Cer1 | cerberus 1 homolog (Xenopus laevis) | 12622 |
| MY204 | Chaf1b | chromatin assembly factor 1, subunit B (p60) | 110749 |
| MH977 | Chat | choline acetyltransferase | 12647 |
| MH978 | Chd2 | chromodomain helicase DNA binding protein 2 | 244059 |
| MY274 | Chodl | chondrolectin | 246048 |
| MH465 | Chrd | chordin | 12667 |
| MH240 | Chrm3 | cholinergic receptor, muscarinic 3, cardiac | 12671 |
| MH241 | Chrm4 | cholinergic receptor, muscarinic 4 | 12672 |
| MH442 | Chrna4 | Cholinergic Receptor nicotinic a-polypeptide4, Chrna4 | 11438 |
| MH112 | Chrnb2 | cholinergic receptor, nicotinic, beta polypeptide 2 (neuronal) | 11444 |
| MH242 | Chrnb4 | cholinergic receptor, nicotinic, beta polypeptide 4 | 108015 |
| MH119 | Chst2 | carbohydrate sulfotransferase 2 | 54371 |
| MH786 | Cited2 | Cbp/p300-interacting transactivator, with Glu/Asp-rich carboxy-terminal domain, 2 | 17684 |
| MH718 | Ckmt1 | creatine kinase, mitochondrial 1, ubiquitous | 12716 |
| MY213 | Cldn8 | Claudin 8 | 54420 |
| MH1002 | Clock | circadian locomoter output cycles kaput | 12753 |
| MH787 | Clu | clusterin | 12759 |
| MH153 | Cnih | cornichon homolog_Drosophila | 12793 |
| MH736 | Cnih3 | cornichon homolog 3 (Drosophila) | 72978 |
| MH744 | Cnil | cornichon-like (Drosophila) | 12794 |
| MH99 | Cnr1 | cannabinoid receptor 1 (brain) | 12801 |
| MH468 | Cntf | ciliary neurotrophic factor | 12803 |
| ST72 | Cntn1 | contactin 1 | 12805 |
| ST67 | Cntn3 | contactin 3 | 18488 |
| ST63 | Cntn6 | contactin 6 | 53870 |
| MY282 | Col18a1 | procollagen, type XVIII, alpha 1 | 12822 |
| HD18 | Col4a1 | procollagen, type IV, alpha 1 | 12826 |
| MY283 | Col6a1 | procollagen, type VI, alpha 1 | 12833 |
| MH788 | Comt | catechol-O-methyltransferase | 12846 |
| HD68 | Copg | coatomer protein complex, subunit gamma | 54161 |
| MH745 | Cpe | carboxypeptidase E | 12876 |
| MH444 | Cpsf5 | cleavage and polyadenylation specific factor 5 | 68219 |
| MH509 | Cpt1a | carnitine palmitolyltransferase 1, liver | 12894 |
| MH618 | Cpt1c | carnitine palmitoyltransferase 1c | 78070 |
| MH607 | Crabp1 | cellular retinoic acid binding protein I | 12903 |
| MH960 | Crb1 | crumbs homolog 1 (Drosophila) | 170788 |
| MH1022 | Creb1 | cAMP responsive element binding protein 1 | 12912 |
| DA117 | Crx | cone-rod homeobox containing gene | 12951 |
| MH979 | Cry2 | cryptochrome 2 (photolyase-like) | 12953 |
| MY214 | Cryaa | crystallin, alpha A | 12954 |
| MY215 | Cryzl1 | crystallin, zeta (quinone reductase)-like 1 | 66609 |
| MH953 | Csl | citrate synthase like | 71832 |
| MH1023 | Csnb | casein beta | 12991 |
| HD13 | Csrp1 | cysteine and glycine-rich protein 1 | 13007 |
| MY210 | Cstb | cystatin B | 13014 |
| MH961 | Ctsl | cathepsin L | 13039 |
| MH511 | Cutl1 | cut-like 1 (Drosophila) | 13047 |
| MY234 | Cxadr | coxsackievirus and adenovirus receptor | 13052 |
| MH970 | Cxcl10 | chemokine (C-X-C motif) ligand 10 | 15945 |
| MH428 | Cxcl12 | chemokine (C-X-C motif) ligand 12 | 20315 |
| MH1254 | Cxcl9 | chemokine (C-X-C motif) ligand 9 | 17329 |
| MH826 | Cyp26a1 | cytochrome P450, family 26, subfamily a, polypeptide 1 | 13082 |
| MH844 | Cyp26b1 | cytochrome P450, family 26, subfamily b, polypeptide 1 | 232174 |
| MH1255 | Cyp4f15 | cytochrome P450, family 4, subfamily f, polypeptide 15 | 106648 |
| MH1099 | Cyr61 | cysteine rich protein 61 | 16007 |
| MH373 | D10Jhu81e | DNA segment, Chr 10, Johns Hopkins University 81 expressed | 28295 |
| MH900 | D10Wsu102e | DNA segment, Chr 10, Wayne State University 102, expressed | 28109 |
| MY200 | D16Ertd472e | DNA segment, Chr 16, ERATO Doi 472, expressed | 67102 |
| ST75 | D930015E06Rik | RIKEN cDNA D930015E06 gene | 229473 |
| MH356 | Dab1 | disabled homolog 1 (Drosophila) | 13131 |
| MH677 | Dazap1 | DAZ associated protein 1 | 70248 |
| MH827 | Dbh | dopamine beta hydroxylase | 13166 |
| MH1003 | Dbp | D site albumin promoter binding protein | 13170 |
| MH1185 | Dcn | decorin | 13179 |
| MH1324 | Dcx | doublecortin | 13193 |
| MH828 | Ddc | dopa decarboxylase | 13195 |
| MH790 | Ddx3 | DEAD/H (Asp-Glu-Ala-Asp/His) box polypeptide 3, X-linked | 13205 |
| MH513 | Ddx5 | DEAD/H (Asp-Glu-Ala-Asp/His) box polypeptide 5 | 13207 |
| MH720 | Dgkg | diacylglycerol kinase, gamma | 110197 |
| MH547 | Dio2 | deiodinase, iodothyronine, type II | 13371 |
| MY227 | Dip2 | disco interacting protein 2 homolog (Drosophila) | 64451 |
| MH621 | Dkk1 | dickkopf homolog 1 (Xenopus laevis) | 13380 |
| MH622 | Dkk2 | dickkopf homolog 2 (Xenopus laevis) | 56811 |
| MH676 | Dkk3 | dickkopf homolog 3 (Xenopus laevis) | 50781 |
| MH746 | Dkk4 | dickkopf homolog 4 (Xenopus laevis) | 234130 |
| MH962 | Dld | dihydrolipoamide dehydrogenase | 13382 |
| HD24 | Dll1 | delta-like 1 (Drosophila) | 13388 |
| MH948 | Dlst | dihydrolipoamide S-succinyltransferase (E2 component of 2-oxo-glutarate complex) | 78920 |
| HD57 | Dlx1 | distal-less homeobox 1 | 13390 |
| MH445 | Dlx2 | distal-less homeobox 2 | 13392 |
| MH564 | Dlx3 | distal-less homeobox 3 | 13393 |
| MH747 | Dlx4 | distal-less homeobox 4 | 13394 |
| MH478 | Dlx5 | distal-less homeobox 5 | 13395 |
| MH614 | Dlx6 | distal-less homeobox 6 | 13396 |
| MH748 | Dnajb9 | DnaJ (Hsp40) homolog, subfamily B, member 9 | 27362 |
| MY311 | Donson | downstream neighbor of SON | 60364 |
| MH843 | Dpf2 | D4, zinc and double PHD fingers family 2 | 19708 |
| MH829 | Dpysl4 | dihydropyrimidinase-like 4 | 26757 |
| MH1044 | Drd1a | dopamine receptor D1A | 13488 |
| MH1045 | Drd2 | dopamine receptor 2 | 13489 |
| MH421 | Drd4 | dopamine Receptor 4 | 13491 |
| MY209 | Dscam | Down syndrome cell adhesion molecule | 13508 |
| MY300 | Dscr1 | Down syndrome critical region homolog 1 (human) | 54720 |
| MY197 | Dscr2 | Down syndrome critical region homolog 2 (human) | 56088 |
| MH389 | Dscr3 | Down syndrome critical region gene 3 | 13185 |
| MY253 | Dscr5 | Down syndrome critical region homolog 5 (human) | 56176 |
| MY292 | Dscr6 | Down syndrome critical region homolog 6 (human) | 170765 |
| MY284 | Dtx1 | deltex 1 homolog (Drosophila) | 14357 |
| HD20 | Dusp16 | dual specificity phosphatase 16 | 70686 |
| MY241 | Dyrk1a | dual-specificity tyrosine-(Y)-phosphorylation regulated kinase 1a | 13548 |
| MH155 | E130016E03Rik | RIKEN cDNA E130016E03 gene | 320890 |
| MH514 | E2f1 | E2F transcription factor 1 | 13555 |
| MH1024 | E2f2 | E2F transcription factor 2 | 242705 |
| MH623 | E2f3 | E2F transcription factor 3 | 13557 |
| MH565 | E2f4 | E2F transcription factor 4 | 104394 |
| MH566 | E2f5 | E2F transcription factor 5 | 13559 |
| MH567 | E2f6 | E2F transcription factor 6 | 50496 |
| MH470 | E4f1 | E4F transcription factor 1 | 13560 |
| MH1369 | Ebf1 | early B-cell factor 1 | 13591 |
| HD65 | Efna1 | ephrin A1 | 13636 |
| MH1240 | Efna2 | ephrin A2 | 13637 |
| HD66 | Efna3 | ephrin A3 | 13638 |
| HD72 | EfnA4 | ephrin A4 | 13639 |
| HD70 | Efna5 | ephrin A5 | 13640 |
| MH548 | Efnb1 | ephrin B1 | 13641 |
| MH791 | Efnb3 | ephrin B3 | 13643 |
| MH981 | Ei24 | etoposide induced 2.4 mRNA | 13663 |
| HD27 | Eif2ak3 | eukaryotic translation initiation factor 2 alpha kinase 3 | 13666 |
| DA103 | Elf1 | E74-like factor 1 | 13709 |
| DA104 | Elf2 | E74-like factor 2 | 69257 |
| DA105 | Elf4 | E74-like factor 4 (ets domain transcription factor) | 56501 |
| DA129 | Elk1 | ELK1, member of ETS oncogene family | 13712 |
| DA106 | Elk3 | ELK3, member of ETS oncogene family | 13713 |
| MH749 | Enc1 | ectodermal-neural cortex 1 | 13803 |
| HD79 | Entpd1 | ectonucleoside triphosphate diphosphohydrolase 1 | 12495 |
| MH598 | Eomes | eomesodermin homolog (Xenopus laevis) | 13813 |
| MH423 | Epha4 | Eph receptor A4 | 13838 |
| HD44 | Epha7 | Eph receptor A7 | 13841 |
| HD42 | Eps8l3 | ESP8-like 3 | 99662 |
| MH726 | Erdr1 | erythroid differentiation regulator 1 | 170942 |
| DA107 | Erf | Ets2 repressor factor | 13875 |
| DA123 | Erg | avian erythroblastosis virus E-26 (v-ets) oncogene related | 13876 |
| MH568 | Erh | enhancer of rudimentary homolog (Drosophila) | 13877 |
| MH569 | Esr1 | estrogen receptor 1 (alpha) | 13982 |
| MH792 | Esrra | estrogen related receptor, alpha | 26379 |
| MH793 | Esrrb | estrogen related receptor, beta | 26380 |
| DA108 | Ets1 | E26 avian leukemia oncogene 1, 5' domain | 23871 |
| DA79 | Etv3 | ets variant gene 3 | 27049 |
| DA109 | Etv6 | ets variant gene 6 (TEL oncogene) | 14011 |
| MH570 | Evi1 | ecotropic viral integration site 1 | 14013 |
| MH1241 | Evi2a | ecotropic viral integration site 2a | 14017 |
| MH794 | Ewsh | Ewing sarcoma breakpoint region 1 | 14030 |
| MH186 | Exo1 | exonuclease 1 | 26909 |
| ST76 | Extl3 | exostoses (multiple)-like 3 | 54616 |
| DC38 | Fabp3 | fatty acid binding protein 3, muscle and heart | 14077 |
| MH115 | Fbxo2 | F-box only protein 2 | 230904 |
| DA71 | Ferd3l | Fer3-like (Drosophila) | 114712 |
| FG50 | Fgf1 | fibroblast growth factor 1 | 14164 |
| MH359 | Fgf10 | fibroblast growth factor 10 | 14165 |
| FG25 | Fgf11 | fibroblast growth factor 11 | 14166 |
| FG53 | Fgf12 | fibroblast growth factor 12 | 14167 |
| FG21 | Fgf13 | fibroblast growth factor 13 | 14168 |
| FG54 | Fgf14 | fibroblast growth factor 14 | 14169 |
| FG49 | Fgf15 | fibroblast growth factor 15 | 14170 |
| FG42 | Fgf16 | fibroblast growth factor 16 | 80903 |
| FG51 | Fgf17 | fibroblast growth factor 17 | 14171 |
| FG55 | Fgf18 | fibroblast growth factor 18 | 14172 |
| FG39 | Fgf2 | fibroblast growth factor 2 | 14173 |
| FG40 | Fgf20 | fibroblast growth factor 20 | 80857 |
| FG41 | Fgf21 | fibroblast growth factor 21 | 56636 |
| FG32 | Fgf22 | fibroblast growth factor 22 | 67112 |
| FG43 | Fgf23 | fibroblast growth factor 23 | 64654 |
| FG24 | Fgf3 | fibroblast growth factor 3 | 14174 |
| FG44 | Fgf4 | fibroblast growth factor 4 | 14175 |
| FG26 | Fgf5 | fibroblast growth factor 5 | 14176 |
| FG46 | Fgf6 | fibroblast growth factor 6 | 14177 |
| FG58 | Fgf7 | fibroblast growth factor 7 | 14178 |
| MH358 | Fgf8 | fibroblast growth factor 8 | 14179 |
| FG52 | Fgf9 | fibroblast growth factor 9 | 14180 |
| HD55 | Fgfbp1 | fibroblast growth factor binding protein 1 | 14181 |
| FG34 | Fgfr1 | fibroblast growth factor receptor 1 | 14182 |
| FG35 | Fgfr2 | fibroblast growth factor receptor 2 | 14183 |
| FG36 | Fgfr3 | fibroblast growth factor receptor 3 | 14184 |
| FG37 | Fgfr4 | fibroblast growth factor receptor 4 | 14186 |
| MH954 | Fh1 | fumarate hydratase 1 | 14194 |
| MH1102 | Fhl1 | four and a half LIM domains 1 | 14199 |
| MH154 | Fjx1 | Four jointed box | 14221 |
| MH795 | Fkbp3 | FK506 binding protein 3 | 30795 |
| DA110 | Fli1 | Friend leukemia integration 1 | 14247 |
| MH818 | Flot1 | flotillin 1 | 14251 |
| MH751 | Fnbp3 | formin binding protein 3 | 56194 |
| MH982 | Fos | FBJ osteosarcoma oncogene | 14281 |
| MH721 | Fosl2 | fos-like antigen 2 | 14284 |
| MH864 | Foxa1 | forkhead box A1 | 15375 |
| MH515 | Foxa2 | forkhead box A2 | 15376 |
| HD30 | Foxb1 | forkhead box B1 | 64290 |
| MH1103 | Foxb2 | forkhead box B2 | 14240 |
| MH516 | Foxd1 | forkhead box D1 | 15229 |
| MH620 | Foxd3 | forkhead box D3 | 15221 |
| MH224 | Foxg1 | forkhead box G1 | 15228 |
| MH619 | Foxi1 | forkhead box I1 | 14233 |
| MH517 | Foxj2 | forkhead box J2 | 60611 |
| MH518 | Foxl1 | forkhead box L1 | 14241 |
| MH632 | Foxm1 | forkhead box M1 | 14235 |
| MH1256 | Frat2 | frequently rearranged in advanced T-cell lymphomas 2 | 212398 |
| ST31 | Frem2 | Fras1 related extracellular matrix protein 2 | 242022 |
| MH678 | Frzb | frizzled-related protein | 20378 |
| MY242 | Ftcd | formiminotransferase cyclodeaminase | 14317 |
| MH865 | Ftsj1 | Ftsj homolog (E. coli) | 54632 |
| MH679 | Fzd1 | frizzled homolog 1 (Drosophila) | 14362 |
| MH624 | Fzd10 | frizzled homolog 10 (Drosophila) | 93897 |
| MH866 | Fzd2 | frizzled homolog 2 (Drosophila) | 57265 |
| MH732 | Fzd3 | frizzled homolog 3 (Drosophila) | 14365 |
| MH752 | Fzd4 | frizzled homolog 4 (Drosophila) | 14366 |
| MH830 | Fzd5 | frizzled homolog 5 (Drosophila) | 14367 |
| MH753 | Fzd6 | frizzled homolog 6 (Drosophila) | 14368 |
| MH680 | Fzd7 | frizzled homolog 7 (Drosophila) | 14369 |
| MH912 | Fzd8 | frizzled homolog 8 (Drosophila) | 14370 |
| MH796 | G3bp | Ras-GTPase-activating protein SH3-domain binding protein | 27041 |
| MY285 | Gabpa | GA repeat binding protein, alpha | 14390 |
| MH890 | Gabrb1 | gamma-aminobutyric acid (GABA-A) receptor, subunit beta 1 | 14400 |
| MH225 | Gabrb2 | gamma-aminobutyric acid (GABA-A) receptor, subunit beta 2 | 14401 |
| MH797 | Gabre | gamma-aminobutyric acid (GABA-A) receptor, subunit epsilon | 14404 |
| MH448 | Gad2 | Glutamic acid decarboxylase 2 | 14417 |
| MH557 | Gadd45g | growth arrest and DNA-damage-inducible 45 gamma | 23882 |
| MH983 | Gan | giant axonal neuropathy | 209239 |
| MY286 | Gart | phosphoribosylglycinamide formyltransferase | 14450 |
| MH360 | Gas1 | growth arrest specific 1 | 14451 |
| MH173 | Gas5 | Growth arrest specific 5 | 14455 |
| MH831 | Gata1 | GATA binding protein 1 | 14460 |
| MH571 | Gata2 | GATA binding protein 2 | 14461 |
| MH754 | Gata3 | GATA binding protein 3 | 14462 |
| MH1025 | Gbp1 | guanylate nucleotide binding protein 1 | 14468 |
| MH971 | Gbp2 | guanylate nucleotide binding protein 2 | 14469 |
| MH867 | Gbx2 | gastrulation brain homeobox 2 | 14472 |
| DC42 | Gdi3 | guanosine diphosphate (GDP) dissociation inhibitor 3 | 14569 |
| MH1243 | Gfap | glial fibrillary acidic protein | 14580 |
| MH1153 | Gfi1 | growth factor independent 1 | 14581 |
| MH692 | Gh | growth hormone | 14599 |
| MH170 | Ghitm | Growth hormone inducible transmembrane protein | 66092 |
| MH1104 | Ghrh | growth hormone releasing hormone | 14601 |
| MH779 | Ghrl | ghrelin | 58991 |
| HD12 | Gja1 | gap junction membrane channel protein alpha 1 | 14609 |
| HD21 | Gjb2 | gap junction membrane channel protein beta 2 | 14619 |
| MH486 | Gjb5 | gap junction membrane channel protein beta 5 | 14622 |
| MH1026 | Gli3 | GLI-Kruppel family member GLI3 | 14634 |
| MH921 | Glrb | glycine receptor, beta subunit | 14658 |
| MH733 | Gls | glutaminase | 14660 |
| MH832 | Glud1 | glutamate dehydrogenase 1 | 14661 |
| MH777 | Gm623 | Gene model 623, (NCBI) | 268288 |
| MH207 | Gmps | guanine monphosphate synthetase | 229363 |
| MH625 | Gnai2 | guanine nucleotide binding protein, alpha inhibiting 2 | 14678 |
| MH633 | Gnao | guanine nucleotide binding protein, alpha o | 14681 |
| MH480 | Gnas | GNAS (guanine nucleotide binding protein, alpha stimulating) complex locus | 14683 |
| HD76 | Gnat1 | guanine nucleotide binding protein, alpha transducing 1 | 14685 |
| MH634 | Gnb2 | guanine nucleotide binding protein, beta 2 | 14693 |
| MH681 | Golga4 | golgi autoantigen, golgin subfamily a, 4 | 54214 |
| MH922 | Gpr101 | G protein-coupled receptor 101 | 245424 |
| MH799 | Gpr81 | G protein-coupled receptor 81 | 243270 |
| MH800 | Gpr82 | G protein-coupled receptor 82 | 319200 |
| MH801 | Gpr88 | G-protein coupled receptor 88 | 64378 |
| MH833 | Gria1 | glutamate receptor, ionotropic, AMPA1 (alpha 1) | 14799 |
| MY301 | Grik1 | glutamate receptor, ionotropic, kainate 1 | 14805 |
| MH755 | Grin1 | glutamate receptor, ionotropic, NMDA1 (zeta 1) | 14810 |
| MH984 | Grin3b | glutamate receptor, ionotropic, NMDA3B | 170483 |
| MH372 | Grm1 | glutamate receptor, metabotropic 1 | 14816 |
| MH371 | Grm3 | glutamate receptor, metabotropic 3 | 108069 |
| MH722 | Grn | granulin | 14824 |
| MH1087 | Grp | gastrin releasing peptide | 225642 |
| DA111 | Gsc | goosecoid | 14836 |
| MH756 | Gsh1 | genomic screened homeo box 1 | 14842 |
| MH1230 | Gsn | gelsolin | 227753 |
| MH472 | Gtf2h4 | general transcription factor II H, polypeptide 4 | 14885 |
| MH780 | Gtf3c2 | general transcription factor IIIC, polypeptide 2, beta | 71752 |
| MH757 | Gucy1a3 | guanylate cyclase 1, soluble, alpha 3 | 60596 |
| MH845 | Guk1 | guanylate kinase 1 | 14923 |
| HD61 | H2afy | H2A histone family, member Y | 26914 |
| MH519 | Hand1 | heart and neural crest derivatives expressed transcript 1 | 15110 |
| MH834 | Hap1 | huntingtin-associated protein 1 | 15114 |
| MH1004 | Hcrt | hypocretin | 15171 |
| MH438 | Hcrtr1 | hypocretin (orexin) receptor 1 | 230777 |
| MH1692 | Hcrtr2 | hypocretin (orexin) receptor 2 | 57781 |
| HD25 | Hectd2 | HECT domain containing 2 | 226098 |
| MH1028 | Hes2 | hairy and enhancer of split 2 (Drosophila) | 15206 |
| MH1106 | Hes3 | hairy and enhancer of split 3 (Drosophila) | 15207 |
| MH945 | Hes6 | hairy enhancer of split 6, (Drosophila) | 55927 |
| MH1257 | Hes7 | hairy and enhancer of split 7 (Drosophila) | 84653 |
| DA118 | Hesx1 | homeo box gene expressed in ES cells | 15209 |
| MH1046 | Hgf | hepatocyte growth factor | 15234 |
| MH802 | Hip2 | huntingtin interacting protein 2 | 53323 |
| MH1209 | Hipk2 | homeodomain interacting protein kinase 2 | 15258 |
| MY13 | Hist1h2bc | histone 1, H2bc | 68024 |
| MY136 | Hlcs | holocarboxylase synthetase (biotin- [propriony-Coenzyme A-carboxylase (ATP-hydrolysing)] ligase) | 110948 |
| MH520 | Hlf | hepatic leukemia factor | 217082 |
| MY206 | Hmgn1 | high mobility group nucleosomal binding domain 1 | 15312 |
| MH521 | Hnf4 | hepatic nuclear factor 4 | 15378 |
| MH891 | Hnrpdl | heterogeneous nuclear ribonucleoprotein D-like | 50926 |
| MH383 | Hnt | neurotrimin | 235106 |
| MH758 | Homer1 | homer homolog 1 (Drosophila) | 26556 |
| MH731 | Hook3 | hook homolog 3 (Drosophila) | 320191 |
| HD15 | Hoxa2 | homeo box A2 | 15399 |
| MH963 | Hoxa9 | homeo box A9 | 15405 |
| MH394 | Hrmt1L1 | heterogeneous nuclear ribonucleoprotein methyltransferase-like 1 (S. cerevisiae) | 15468 |
| MH1029 | Hsf1 | heat shock factor 1 | 15499 |
| MH1261 | Hsf2 | heat shock factor 2 | 15500 |
| MY287 | Hsf2bp | heat shock transcription factor 2 binding protein | 74377 |
| DC41 | Hspb7 | heat shock protein family, member 7 (cardiovascular) | 29818 |
| DC26 | Hspb8 | heat shock 27kDa protein 8 | 80888 |
| MH1005 | Icmt | isoprenylcysteine carboxyl methyltransferase | 57295 |
| MH374 | Icosl | icos ligand | 50723 |
| MH964 | Idh1 | isocitrate dehydrogenase 1 (NADP+), soluble | 15926 |
| MH965 | Idh3a | isocitrate dehydrogenase 3 (NAD+) alpha | 67834 |
| MH973 | Ifi47 | interferon gamma inducible protein 47 | 15953 |
| MH989 | Ifit1 | interferon-induced protein with tetratricopeptide repeats 1 | 15957 |
| MH1210 | Ifit2 | interferon-induced protein with tetratricopeptide repeats 2 | 15958 |
| MH990 | Ifit3 | interferon-induced protein with tetratricopeptide repeats 3 | 15959 |
| MY302 | Ifnar1 | interferon (alpha and beta) receptor 1 | 15975 |
| MY271 | Ifnar2 | interferon (alpha and beta) receptor 2 | 15976 |
| MY307 | Ifngr2 | interferon gamma receptor 2 | 15980 |
| MH759 | Igbp1 | immunoglobulin (CD79A) binding protein 1 | 18518 |
| MH803 | Igf1 | insulin-like growth factor 1 | 16000 |
| MH523 | Igf2 | insulin-like growth factor 2 | 16002 |
| MH554 | Igfbpl1 | RIKEN cDNA 2810011G06 gene | 75426 |
| MH972 | Igtp | interferon gamma inducible GTPase | 16145 |
| MH1322 | Iigp1 | interferon inducible GTPase 1 | 60440 |
| MY257 | Il10rb | interleukin 10 receptor, beta | 16155 |
| MY258 | Irx1 | Iroquois related homeobox 1 (Drosophila) | 16371 |
| MY264 | Irx2 | Iroquois related homeobox 2 (Drosophila) | 16372 |
| MH342 | Isl1 | ISL1 transcription factor, LIM/homeodomain (islet 1) | 16392 |
| MH406 | Itga3 | integrin alpha 3 | 16400 |
| MH550 | Itga4 | integrin alpha 4 | 16401 |
| MY259 | Itgb2 | integrin beta 2 | 16414 |
| MY7 | Itsn1 | intersectin 1 (SH3 domain protein 1A) | 16443 |
| MH420 | Jam2 | junction adhesion molecule 2 | 67374 |
| MY216 | Jam4 | junction adhesion molecule 4 | 72058 |
| MH846 | Jarid2 | jumonji, AT rich interactive domain 2 | 16468 |
| MH524 | Jun | Jun oncogene | 16476 |
| MH572 | Jund1 | Jun proto-oncogene related gene d1 | 16478 |
| MY308 | Kcne1 | potassium voltage-gated channel, Isk-related subfamily, member 1 | 16509 |
| MY261 | Kcne2 | potassium voltage-gated channel, Isk-related subfamily, gene 2 | 246133 |
| MY207 | Kcnj15 | potassium inwardly-rectifying channel, subfamily J, member 15 | 16516 |
| MY250 | Kcnj6 | potassium inwardly-rectifying channel, subfamily J, member 6 | 16522 |
| MH760 | Kcnn4 | potassium intermediate/small conductance calcium-activated channel, subfamily N, member 4 | 16534 |
| MH483 | Kif11 | kinesin family member 11 | 16551 |
| MH484 | Kif4 | kinesin family member 4 | 16571 |
| MH761 | Kif5a | kinesin family member 5A | 16572 |
| HD48 | Kif5B | kinesin family member 5B | 16573 |
| MH487 | Kifc2 | kinesin family member C2 | 16581 |
| MH166 | Kifc5a | kinesin family member C5A | 94116 |
| HD11 | Kitl | kit ligand | 17311 |
| MH574 | Knsl7 | kinesin-like 7 | 209737 |
| HD64 | Kpna2 | karyopherin (importin) alpha 2 | 16647 |
| MH762 | Kpna4 | karyopherin (importin) alpha 4 | 16649 |
| MH734 | Kremen1 | kringle containing transmembrane protein 1 | 84035 |
| MY251 | Krtap11-1 | keratin associated protein 11-1 | 16693 |
| MH1264 | Krtap12-1 | keratin associated protein 12-1 | 16694 |
| MH763 | L1cam | L1 cell adhesion molecule | 16728 |
| MH575 | Laf4l | lymphoid nuclear protein related to AF4-like | 93736 |
| MH488 | Lamc1 | laminin, gamma 1 | 226519 |
| MH868 | Lancl1 | LanC (bacterial lantibiotic synthetase component C)-like | 14768 |
| DC30 | Ldb3 | LIM domain binding 3 | 24131 |
| MH764 | Lef1 | lymphoid enhancer binding factor 1 | 16842 |
| MH346 | Lhx1 | LIM homeobox protein 1 | 16869 |
| MH353 | Lhx2 | LIM homeobox protein 2 | 16870 |
| MH375 | Lhx3 | LIM homeobox protein 3 | 16871 |
| MH398 | Lhx4 | LIM homeobox protein 4 | 16872 |
| MH345 | Lhx5 | LIM homeobox protein 5 | 16873 |
| MH407 | Lhx6 | LIM homeobox protein 6 | 16874 |
| MH612 | Lhx8 | LIM homeobox protein 8 | 16875 |
| MH399 | Lhx9 | LIM homeobox protein 9 | 16876 |
| MH344 | Limk1 | LIM-domain containing, protein kinase | 16885 |
| MH397 | Lix1 | limb expression 1 homolog (chicken) | 66643 |
| MH835 | Llglh | lethal giant larvae homolog | 16897 |
| MH349 | Lmo1 | LIM domain only 1 | 109594 |
| MH350 | Lmo2 | LIM domain only 2 | 16909 |
| MH1015 | Lmx1b | LIM homeobox transcription factor 1 beta | 16917 |
| MH1325 | LOC114601 | tangerin | 114601 |
| MH1343 | LOC227485 | similar to cadherin 19, type 2 preproprotein | 227485 |
| MH237 | Lpd | lipidosin | 94180 |
| DC49 | Lpl | lipoprotein lipase | 16956 |
| MH431 | Lrig1 | leucine-rich repeats and immunoglobulin-like domains 1 | 16206 |
| ST89 | Lrig2 | leucine-rich repeats and immunoglobulin-like domains 2 | 269473 |
| ST90 | Lrig3 | leucine-rich repeats and immunoglobulin-like domains 3 | 320398 |
| MH913 | Lrp5 | low density lipoprotein receptor-related protein 5 | 16973 |
| MH821 | Lrp6 | low density lipoprotein receptor-related protein 6 | 16974 |
| MY192 | Lrrc3 | leucine-rich repeat-containing 3 | 237387 |
| ST65 | Lsamp | limbic system-associated membrane protein | 268890 |
| MH347 | Lss | lanosterol synthase | 16987 |
| MH804 | Lta4h | leukotriene A4 hydrolase | 16993 |
| MH901 | Ltbp4 | latent transforming growth factor beta binding protein 4 | 108075 |
| MH348 | Lynx1 | Ly6/neurotoxin 1 | 23936 |
| MH974 | Mad | max dimerization protein | 17119 |
| HD8 | Mageh1 | melanoma antigen, family H, 1 | 75625 |
| HD26 | Mansc1 | MANSC domain containing 1 | 67729 |
| MH489 | Map3k7 | mitogen activated protein kinase kinase kinase 7 | 26409 |
| HD39 | Map4k3 | mitogen-activated protein kinase kinase kinase kinase3 (Map4k3) | 225028 |
| MH950 | Mapk6 | mitogen-activated protein kinase 6 | 50772 |
| MH951 | Mapkapk5 | MAP kinase-activated protein kinase 5 | 17165 |
| MH577 | Mapre1 | microtubule-associated protein, RP/EB family, member 1 | 13589 |
| MH409 | Mc4r | melanocortin 4 receptor | 17202 |
| MY294 | Mcm3ap | minichromosome maintenance deficient 3 (S. cerevisiae) associated protein | 54387 |
| MH608 | Mcm5 | minichromosome maintenance deficient 5, cell division cycle 46 (S. cerevisiae) | 17218 |
| MH551 | Mcm7 | mini chromosome maintenance deficient 7 (S. cerevisiae) | 17220 |
| MH966 | Mdh1 | malate dehydrogenase 1, NAD (soluble) | 17449 |
| MH609 | Mdk | midkine | 17242 |
| MH985 | Mecp2 | methyl CpG binding protein 2 | 17257 |
| MH765 | Mef2c | myocyte enhancer factor 2C | 17260 |
| MH578 | Mfng | manic fringe homolog (Drosophila) | 17305 |
| MH1108 | Mglap | matrix gamma-carboxyglutamate (gla) protein | 17313 |
| ST77 | Mid2 | midline 2 | 23947 |
| MH552 | Mif | macrophage migration inhibitory factor | 17319 |
| DA124 | Mixl1 | Mix1 homeobox-like 1 (Xenopus laevis) | 27217 |
| MH610 | Mki67 | antigen identified by monoclonal antibody Ki 67 | 17345 |
| MH490 | Mlp | MARCKS-like protein | 17357 |
| MH408 | Mmp15 | matrix metalloproteinase 15 | 17388 |
| MH611 | Mrg1 | myeloid ecotropic viral integration site-related gene 1 | 17536 |
| MY208 | Mrpl39 | mitochondrial ribosomal protein L39 | 27393 |
| MH980 | Msl2 | male-specific lethal-2 homolog (Drosophila) | 77853 |
| MH437 | Mtf2 | metal response element binding transcription factor 2 | 17765 |
| MH724 | Mtif2 | mitochondrial translational initiation factor 2 | 76784 |
| MH986 | mt-Nd3 | NADH dehydrogenase 3, mitochondrial | 17718 |
| MH636 | Mtpn | myotrophin | 14489 |
| MH869 | Mtx2 | metaxin 2 | 53375 |
| MH208 | Mup2 | major urinary protein 2 | 17841 |
| MY288 | Mx1 | myxovirus (influenza virus) resistance 1 | 17857 |
| MY289 | Mx2 | myxovirus (influenza virus) resistance 2 | 17858 |
| MH766 | Myb | myeloblastosis oncogene | 17863 |
| DC40 | Mybpc3 | myosin binding protein C, cardiac | 17868 |
| MH836 | Myc | myelocytomatosis oncogene | 17869 |
| MH1109 | Myf5 | myogenic factor 5 | 17877 |
| HD59 | Mylip | myosin regulatory light chain interacting protein | 218203 |
| MH1319 | Mylk | myosin, light polypeptide kinase | 107589 |
| DC35 | Myom1 | myomesin 1 | 17929 |
| MH161 | Myst1 | MYST histone acetyltransferase 1 | 67773 |
| MH892 | Nbr1 | neighbor of Brca1 gene 1 | 17966 |
| MY217 | Ncam2 | neural cell adhesion molecule 2 | 17968 |
| MH1244 | Ncor1 | nuclear receptor co-repressor 1 | 20185 |
| MH191 | Ndel1 | nuclear distribution gene E-like homolog 1 (A. nidulans) | 83431 |
| MH837 | Ndrg2 | N-myc downstream regulated gene 2 | 29811 |
| ST61 | Negr1 | neuronal growth regulator 1 | 320840 |
| MH1212 | Neo1 | neogenin | 18007 |
| MH228 | Nes | nestin | 18008 |
| DA125 | Neurod1 | neurogenic differentiation 1 | 18012 |
| DA87 | Neurod4 | neurogenic differentiation 4 | 11923 |
| MH376 | Neurod6 | neurogenic differentiation 6 | 11922 |
| DA72 | Neurog1 | neurogenin 1 | 18014 |
| MH236 | Neurog2 | neurogenin2 | 11924 |
| DA126 | Neurog3 | neurogenin 3 | 11925 |
| MH805 | Nf2 | neurofibromatosis 2 | 18016 |
| MH637 | Nfe2l2 | nuclear, factor, erythroid derived 2, like 2 | 18024 |
| MH638 | Nfia | nuclear factor I/A | 18027 |
| MH725 | Nfib | nuclear factor I/B | 18028 |
| MH639 | Nfix | nuclear factor I/X | 18032 |
| MH526 | Nfkb1 | nuclear factor of kappa light chain gene enhancer in B-cells 1, p105 | 18033 |
| MH413 | Ngfr | nerve growth factor receptor (TNFR superfamily, member 16) | 18053 |
| MH924 | Nkx2-4 | NK2 transcription factor related, locus 4 (Drosophila) | 228731 |
| MH1017 | Nkx2-5 | NK2 transcription factor related, locus 5 (Drosophila) | 18091 |
| MH1031 | Nkx2-6 | NK2 transcription factor related, locus 6 (Drosophila) | 18092 |
| MY186 | Nnp1 | novel nuclear protein 1 | 18114 |
| MH120 | Notch3 | Notch gene homolog 3 (Drosophila) | 18131 |
| MH1214 | Nov | nephroblastoma overexpressed gene | 18133 |
| MH896 | Npepps | aminopeptidase puromycin sensitive | 19155 |
| MH907 | Nphs1 | nephrosis 1 homolog, nephrin (human) | 54631 |
| MH433 | Npy | neuropeptide Y | 109648 |
| MH1245 | Nr0b1 | nuclear receptor subfamily 0, group B, member 1 | 11614 |
| MH1182 | Nr0b2 | nuclear receptor subfamily 0, group B, member 2 | 23957 |
| MH491 | Nr1d2 | nuclear receptor subfamily 1, group D, member 2 | 353187 |
| MH870 | Nr1h4 | nuclear receptor subfamily 1, group H, member 4 | 20186 |
| MH926 | Nr1i3 | nuclear receptor subfamily 1, group I, member 3 | 12355 |
| MH1093 | Nr2c1 | nuclear receptor subfamily 2, group C, member 1 | 22025 |
| MH806 | Nr2e1 | nuclear receptor subfamily 2, group E, member 1 | 21907 |
| MH1160 | Nr2f2 | nuclear receptor subfamily 2, group F, member 2 | 11819 |
| MH446 | Nr2f6 | nuclear receptor subfamily 2, group F, member 6 | 13864 |
| MH807 | Nr3c1 | nuclear receptor subfamily 3, group C, member 1 | 14815 |
| MH1161 | Nr4a2 | nuclear receptor subfamily 4, group A, member 2 | 18227 |
| HD10 | Nr5a1 | nuclear receptor subfamily 5, group A, member 1 | 26423 |
| MY211 | Nrip1 | nuclear receptor interacting protein 1 | 268903 |
| MH1007 | Nrtn | neurturin | 18188 |
| MH492 | Nsep1 | nuclease sensitive element binding protein 1 | 22608 |
| MH579 | Ntan1 | N-terminal Asn amidase | 18203 |
| MH418 | Ntf3 | neurotrophin 3 | 18205 |
| MH541 | Ntf5 | neurotrophin 5 | 78405 |
| MH132 | Ntrk2 | neurotrophic tyrosine kinase, receptor, type 2 | 18212 |
| MH391 | Ntrk3 | neurotrophic tyrosine kinase, receptor, type 3 | 18213 |
| MH1162 | Nudc | nuclear distribution gene C homolog (Aspergillus) | 18221 |
| MH1008 | Nup160 | nucleoporin 160 | 59015 |
| MH1187 | Nup62 | nucleoporin 62 | 18226 |
| MH1246 | Nvl | nuclear VCP-like | 67459 |
| MH871 | Oaz2 | ornithine decarboxylase antizyme 2 | 18247 |
| ST69 | Odz1 | odd Oz/ten-m homolog 1 (Drosophila) | 23963 |
| ST48 | Odz2 | odd Oz/ten-m homolog 2 (Drosophila) | 23964 |
| ST49 | Odz3 | odd Oz/ten-m homolog 3 (Drosophila) | 23965 |
| ST50 | Odz4 | odd Oz/ten-m homolog 4 (Drosophila) | 23966 |
| DA130 | Og9x | OG9 homeobox gene | 18292 |
| MH927 | Ogt | O-linked N-acetylglucosamine (GlcNAc) transferase (UDP-N-acetylglucosamine:polypeptide-N-acetylglucosaminyl transferase) | 108155 |
| MY245 | Olig2 | oligodendrocyte transcription factor 2 | 50913 |
| MH1032 | Opn4 | opsin 4 (melanopsin) | 30044 |
| MH1259 | Optn | optineurin | 71648 |
| MY265 | ORF21 | open reading frame 21 | 224440 |
| MY272 | ORF5 | open reading frame 5 | 53858 |
| MY218 | ORF63 | open reading frame 63 | 224419 |
| MY143 | ORF9 | open reading frame 9 | 52793 |
| MH928 | Otp | orthopedia homolog (Drosophila) | 18420 |
| DA73 | Otx1 | orthodenticle homolog 1 (Drosophila) | 18423 |
| DA99 | Otx2 | orthodenticle homolog 2 (Drosophila) | 18424 |
| DA70 | Otx3 | orthodenticle homolog 3 (Drosophila) | 140477 |
| MH798 | Oxgr1 | oxoglutarate (alpha-ketoglutarate) receptor 1 | 239283 |
| MH1227 | Oxtr | oxytocin receptor | 18430 |
| MH471 | Pafah1b1 | platelet-activating factor acetylhydrolase, isoform 1b, beta1 subunit | 18472 |
| MH1009 | Papola | poly (A) polymerase alpha | 18789 |
| MH1232 | Park2 | parkin | 50873 |
| MH1314 | Parva | parvin, alpha | 57342 |
| MH1315 | Parvb | parvin, beta | 170736 |
| MH1316 | Parvg | parvin, gamma | 64099 |
| DA88 | Pax3 | paired box gene 3 | 18505 |
| MH454 | Pax6 | paired box gene 6 | 18508 |
| DA74 | Pax7 | paired box gene 7 | 18509 |
| MH351 | Pcbp3 | poly(rC) binding protein 3 | 59093 |
| HD22 | Pcdh9 | protocadherin 9 | 211712 |
| MH992 | Pcdha10 | protocadherin alpha 10 | 12943 |
| MH994 | Pcdhga12 | protocadherin gamma subfamily A, 12 | 93724 |
| MY295 | Pcnt2 | pericentrin 2 | 18541 |
| MY252 | Pcp4 | Purkinje cell protein 4 | 18546 |
| HD35 | Pcsk1 | proprotein convertase subtilisin/kexin type 1 | 18548 |
| MH767 | Pcsk1n | proprotein convertase subtilisin/kexin type 1 inhibitor | 30052 |
| MH527 | Pde1a | phosphodiesterase 1A, calmodulin-dependent | 18573 |
| MH601 | Pde1b | phosphodiesterase 1B, Ca2+-calmodulin dependent | 18574 |
| MH905 | Pde1c | phosphodiesterase 1C | 18575 |
| MH617 | Pde4a | phosphodiesterase 4A, cAMP specific | 18577 |
| MH602 | Pde4b | phosphodiesterase 4B, cAMP specific | 18578 |
| MH626 | Pde4d | phosphodiesterase 4D, cAMP specific | 238871 |
| MY201 | Pde9a | phosphodiesterase 9A | 18585 |
| MH1215 | Pdgfra | platelet derived growth factor receptor, alpha polypeptide | 18595 |
| MH967 | Pdxk | pyridoxal (pyridoxine, vitamin B6) kinase | 216134 |
| MH159 | Pelo | pelota homolog (Drosophila) | 105083 |
| DA131 | Pem | placentae and embryos oncofetal gene | 18617 |
| MH1216 | Penk1 | preproenkephalin 1 | 18619 |
| MH450 | Per1 | period homolog 1 (Drosophila) | 18626 |
| MH136 | Per2 | period homolog 2 (Drosophila) | 18627 |
| MH451 | Pex14 | peroxisomal biogenesis factor 14 | 56273 |
| HD38 | Pfkfb2 | 6-phosphofructo-2-kinase/fructose-2,6-biphosphatase 2 | 18460 |
| MH1049 | Pfkfb3 | 6-phosphofructo-2-kinase/fructose-2,6-biphosphatase 3 | 170768 |
| MH395 | Pfkl | phosphofructokinase, liver, B-type | 18641 |
| MH872 | Pgrmc1 | progesterone receptor membrane component 1 | 53328 |
| MH447 | Phc1 | early development regulator 1 (homolog of polyhomeotic 1) | 13619 |
| MH1233 | Phox2a | paired-like homeobox 2a | 11859 |
| MH1217 | Phox2b | paired-like homeobox 2b | 18935 |
| MH528 | Pip5k2c | phosphatidylinositol-4-phosphate 5-kinase, type II, gamma | 117150 |
| DA77 | Pitx1 | paired-like homeodomain transcription factor 1 | 18740 |
| HD52 | Pitx2 | paired-like homeodomain transcription factor 2 | 18741 |
| DA119 | Pitx3 | paired-like homeodomain transcription factor 3 | 18742 |
| MH580 | Pkia | protein kinase inhibitor, alpha | 18767 |
| MY202 | Pknox1 | Pbx/knotted 1 homeobox | 18771 |
| HD53 | Pla2g12 | phospholipase A2, group XIIA | 66350 |
| MH838 | Pld3 | phospholipase D3 | 18807 |
| DC29 | Pln | phospholamban | 18821 |
| MH581 | Plp1 | proteolipid protein (myelin) 1 | 18823 |
| MH1094 | Pltp | phospholipid transfer protein | 18830 |
| ST57 | Plxna3 | plexin A3 | 18846 |
| ST58 | Plxnb1 | plexin B1 | 235611 |
| ST51 | Plxnb2 | Plexin b2 | 140570 |
| ST82 | Plxnb3 | plexin B3 | 140571 |
| ST66 | Plxnc1 | plexin C1 | 54712 |
| MH929 | Pmaip1 | phorbol-12-myristate-13-acetate-induced protein 1 | 58801 |
| MH873 | Pnck | pregnancy upregulated non-ubiquitously expressed CaM kinase | 93843 |
| MH931 | Pnrc1 | proline-rich nuclear receptor coactivator 1 | 108767 |
| MY306 | Pofut2 | protein O-fucosyltransferase 2 | 80294 |
| MH410 | Pomc1 | pro-opiomelanocortin-alpha | 18976 |
| MH683 | Pou2f1 | POU domain, class 2, transcription factor 1 | 18986 |
| MH930 | Pou3f4 | POU domain, class 3, transcription factor 4 | 18994 |
| MH455 | Ppap2a | phosphatic acid phosphatase 2a | 19012 |
| MH816 | Ppara | peroxisome proliferator activated receptor alpha | 19013 |
| MH1033 | Ppard | peroxisome proliferator activator receptor delta | 19015 |
| MH815 | Pparg | peroxisome proliferator activated receptor gamma | 19016 |
| MH600 | Ppil4 | peptidylprolyl isomerase (cyclophilin)-like 4 | 67418 |
| MH894 | Pqbp1 | polyglutamine binding protein 1 | 54633 |
| MH529 | Prc1 | protein regulator of cytokinesis 1 | 233406 |
| DC44 | Prdx1 | peroxiredoxin 1 | 18477 |
| MH599 | Prim1 | DNA primase, p49 subunit | 19075 |
| MH999 | Prkag2 | protein kinase, AMP-activated, gamma 2 non-catalytic subunit | 108099 |
| MH874 | Prkca | protein kinase C, alpha | 18750 |
| MH839 | Prkcz | protein kinase C, zeta | 18762 |
| DA120 | Prrx1 | paired related homeobox 1 | 18933 |
| DA113 | Prrx2 | paired related homeobox 2 | 20204 |
| MY194 | Prss7 | protease, serine, 7 (enterokinase) | 19146 |
| MH582 | Psap | prosaposin | 19156 |
| MH469 | Pscdbp | pleckstrin homology, Sec7 and coiled-coil domains, binding protein | 227929 |
| MH840 | Psen1 | presenilin 1 | 19164 |
| DA92 | Psx2 | placenta specific homeobox 2 | 104384 |
| HD33 | Ptgds | prostaglandin D2 synthetase (brain) | 19215 |
| ST52 | Ptprd | protein tyrosine phosphatase, receptor type, D | 19266 |
| ST59 | Ptprm | protein tyrosine phosphatase, receptor type, M | 19274 |
| ST60 | Ptprt | protein tyrosine phosphatase, receptor type, T | 19281 |
| MH427 | Ptpru | protein tyrosine phosphatase, receptor type, U | 19273 |
| MY189 | Pttg1ip | pituitary tumor-transforming 1 interacting protein | 108705 |
| MH684 | Pvalb | parvalbumin | 19293 |
| MY260 | Pwp2h | PWP2 (periodic tryptophan protein) homolog, yeast | 110816 |
| MH841 | Rab1 | RAB1, member RAS oncogene family | 19324 |
| MH920 | Rab32 | RAB32, member RAS oncogene family | 67844 |
| MH895 | Rad23b | RAD23b homolog (S. cerevisiae) | 19359 |
| MH1242 | Rai16 | retinoic acid induced 16 | 239170 |
| MH842 | Rangap1 | RAN GTPase activating protein 1 | 19387 |
| MH1276 | Rara | retinoic acid receptor, alpha | 19401 |
| MH1115 | Rarg | retinoic acid receptor, gamma | 19411 |
| MH1034 | Rasd1 | RAS, dexamethasone-induced 1 | 19416 |
| HD80 | Rasgrf1 | RAS protein-specific guanine nucleotide-releasing factor 1 | 19417 |
| DA121 | Rax | retina and anterior neural fold homeobox | 19434 |
| HD75 | Rbbp6 | retinoblastoma binding protein 6 | 19647 |
| MY219 | Rbm11 | RNA binding motif protein 11 | 224344 |
| MH172 | Rbm4 | RNA binding motif protein 4 | 19653 |
| MH925 | Rel | reticuloendotheliosis oncogene | 19696 |
| MH380 | Reln | reelin | 19699 |
| MH696 | Rfx1 | regulatory factor X, 1 (influences HLA class II expression) | 19724 |
| MH914 | Rhpn2 | rhophilin, Rho GTPase binding protein 2 | 52428 |
| MH212 | Rnf11 | ring finger protein11 | 29864 |
| MH463 | Rnf128 | ring finger protein 128 | 66889 |
| MH151 | Rnf149 | ring finger protein 149 | 67702 |
| MH674 | Ror1 | receptor tyrosine kinase-like orphan receptor 1 | 26563 |
| MH768 | Rora | RAR-related orphan receptor alpha | 19883 |
| MH414 | Rorb | RAR-related orphan receptor beta | 225998 |
| MH875 | Rpn2 | ribophorin II | 20014 |
| MY155 | Rps5 | ribosomal protein S6 kinase, polypeptide 5 | 20103 |
| MH915 | Rps6ka2 | ribosomal protein S6 kinase, polypeptide 2 | 20112 |
| MH906 | Rps6ka5 | RIKEN cDNA 3110005L17 gene | 73086 |
| MH876 | Rtn2 | reticulon 2 (Z-band associated protein) | 20167 |
| DA89 | Runx1 | runt related transcription factor 1 | 12394 |
| DA90 | Runx2 | runt related transcription factor 2 | 12393 |
| DA91 | Runx3 | runt related transcription factor 3 | 12399 |
| MH1035 | Rxra | retinoid X receptor alpha | 20181 |
| MH1036 | Rxrb | retinoid X receptor beta | 20182 |
| MH697 | Rxrg | retinoid X receptor gamma | 20183 |
| ST88 | Ryk | receptor-like tyrosine kinase | 20187 |
| MH932 | S100a10 | S100 calcium binding protein A10 (calpactin) | 20194 |
| MY23 | S100b | S100 protein, beta polypeptide, neural | 20203 |
| MY296 | Samsn1 | SAM domain, SH3 domain and nuclear localisation signals, 1 | 67742 |
| MH530 | Satb1 | special AT-rich sequence binding protein 1 | 20230 |
| MH877 | Scg2 | secretogranin II | 20254 |
| MH412 | Sema3a | sema domain, immunoglobulin domain (Ig), short basic domain, secreted, (semaphorin) 3A | 20346 |
| MH128 | Sema3b | sema domain, immunoglobulin domain (Ig), short basic domain, secreted, (semaphorin) 3B | 20347 |
| MH584 | Sema3c | sema domain, immunoglobulin domain (Ig), short basic domain, secreted, (semaphorin) 3C | 20348 |
| ST68 | Sema4f | sema domain, immunoglobulin domain (Ig), TM domain, and short cytoplasmic domain | 20355 |
| ST55 | Sema5a | sema domain, seven thrombospondin repeats (type 1 and type 1-like), transmembrane domain (TM) and short cytoplasmic domain, (semaphorin) 5A | 20356 |
| ST71 | Sema5b | sema domain, seven thrombospondin repeats (type 1 and type 1-like), transmembrane domain (TM) and short cytoplasmic domain, (semaphorin) 5B | 20357 |
| ST78 | Sema7a | sema domain, immunoglobulin domain (Ig), and GPI membrane anchor, (semaphorin) 7A | 20361 |
| MH585 | Sept5 | septin 5 | 18951 |
| MH640 | Sept7 | septin 7 | 235072 |
| MH1163 | Serpinf1 | serine (or cysteine) proteinase inhibitor, clade F, member 1 | 20317 |
| MH396 | Setbp1 | SET binding protein 1 | 240427 |
| MH531 | Sfn | stratifin | 55948 |
| DA114 | Sfpi1 | SFFV proviral integration 1 | 20375 |
| MH555 | Sfrp1 | secreted frizzled-related sequence protein 1 | 20377 |
| MH129 | Sfrp2 | secreted frizzled-related sequence protein 2 | 20319 |
| MH698 | Sfrp4 | secreted frizzled-related sequence protein 4 | 20379 |
| MH917 | Sfrp5 | secreted frizzled-related sequence protein 5 | 54612 |
| MY279 | Sfrs15 | splicing factor, arginine/serine-rich 15 | 224432 |
| HD32 | Sgk3 | serum/glucocorticoid regulated kinase 3 | 170755 |
| MY11 | Sh3bgr | SH3-binding domain glutamic acid-rich protein | 50795 |
| MH878 | Shfdg1 | split hand/foot deleted gene 1 | 20422 |
| MH459 | Shh | sonic hedgehog | 20423 |
| MH1018 | Shhrs | sonic hedgehog regulated sequence | 320038 |
| MH558 | Sil | Tal1 interrupting locus | 20460 |
| MY220 | Sim2 | single-minded homolog 2 (Drosophila) | 20465 |
| MH1147 | Skiip | SKI interacting protein | 66354 |
| MH995 | Sla | src-like adaptor | 20491 |
| MH422 | Slc18a2 | solute carrier family 18 (vesicular monoamine), member 2 | 214084 |
| MY266 | Slc19a1 | solute carrier family 19 (sodium/hydrogen exchanger), member 1 | 20509 |
| MH215 | Slc25a3 | solute carrier family 25 (mitochondrial carrier; phosphate carrier), member 3 | 18674 |
| MH452 | Slc30a9 | solute carrier family 30 (zinc transporter), member 9 | 109108 |
| MH425 | Slc36a2 | solute carrier family 36 (proton/amino acid symporter), member 2 | 246049 |
| MH563 | Slc36a4 | solute carrier family 36 (proton/amino acid symporter), member 4 | 234967 |
| MY196 | Slc5a3 | solute carrier family 5 (inositol transporters), member 3 | 53881 |
| MH933 | Slc6a1 | solute carrier family 6 (neurotransmitter transporter, GABA), member 1 | 232333 |
| MH439 | Slc6a3 | solute carrier family 6 (neurotransmitter transporter, dopamine), member 3 | 13162 |
| MH975 | Slfn3 | schlafen 3 | 20557 |
| MH378 | Slit2 | slit homolog 2 (Drosophila) | 20563 |
| MH1222 | Smad1 | MAD homolog 1 (Drosophila) | 17125 |
| MH879 | Smarcb1 | SWI/SNF related, matrix associated, actin dependent regulator of chromatin, subfamily b, member 1 | 20587 |
| DC27 | Smpx | small muscle protein, X-linked | 66106 |
| MH107 | Snca | synuclein, alpha | 20617 |
| MY228 | Snf1lk | SNF1-like kinase | 17691 |
| MY303 | Sod1 | superoxide dismutase 1, soluble | 20655 |
| MH1010 | Son | Son cell proliferation protein | 20658 |
| MH916 | Sox11 | SRY-box containing gene 11 | 20666 |
| MH699 | Sox5 | SRY-box containing gene 5 | 20678 |
| MH1164 | Sox9 | SRY-box containing gene 9 | 20682 |
| DA75 | Spdef | SAM pointed domain containing ets transcription factor | 30051 |
| MH403 | Spg4 | spastic paraplegia 4 homolog (human) | 50850 |
| DA127 | Spic | Spi-C transcription factor (Spi-1/PU.1 related) | 20728 |
| MH1152 | Sprr1a | small proline-rich protein 1A | 20753 |
| MH946 | Srebf1 | sterol regulatory element binding factor 1 | 20787 |
| MH411 | Srgap3 | SLIT-ROBO Rho GTPase activating protein 3 | 259302 |
| MH430 | Sst | somatostatin | 20604 |
| MY195 | Stch | stress 70 protein chaperone, microsome-associated, human homolog | 110920 |
| MH603 | Stmn3 | stathmin-like 3 | 20262 |
| MH588 | Stmn4 | stathmin-like 4 | 56471 |
| MH952 | Suclg1 | succinate-CoA ligase, GDP-forming, alpha subunit | 56451 |
| MY297 | Sumo3 | SMT3 suppressor of mif two 3 homolog 3 (yeast) | 20610 |
| MH1038 | Suv39h2 | suppressor of variegation 3-9 homolog 2 (Drosophila) | 64707 |
| MH363 | Sv2a | synaptic vesicle glycoprotein 2 a | 64051 |
| MH369 | Sv2b | synaptic vesicle glycoprotein 2 b | 64176 |
| MH400 | Sv2c | synaptic vesicle glycoprotein 2c | 75209 |
| MH1016 | Syncrip | synaptotagmin binding, cytoplasmic RNA interacting protein | 56403 |
| MH1249 | Syne1 | synaptic nuclear envelope 1 | 64009 |
| MH1367 | Syne2 | synaptic nuclear envelope 2 | 319565 |
| MH379 | Syngr1 | synaptogyrin 1 | 20972 |
| MH361 | Syngr2 | synaptogyrin 2 | 20973 |
| MH390 | Syngr3 | synaptogyrin 3 | 20974 |
| MY182 | Synj1 | synaptojanin 1 | 104015 |
| MH559 | Syt1 | synaptotagmin 1 | 20979 |
| MH808 | Syt10 | synaptotagmin 10 | 54526 |
| MH493 | Syt11 | synaptotagmin 11 | 229521 |
| MH735 | Syt12 | synaptotagmin 12 | 171180 |
| MH494 | Syt13 | synaptotagmin 13 | 80976 |
| MH495 | Syt2 | synaptotagmin 2 | 20980 |
| MH496 | Syt3 | synaptotagmin 3 | 20981 |
| MH616 | Syt4 | synaptotagmin 4 | 20983 |
| MH615 | Syt5 | synaptotagmin 5 | 53420 |
| MH497 | Syt6 | synaptotagmin 6 | 54524 |
| MH498 | Syt7 | synaptotagmin 7 | 54525 |
| MH231 | Tac1 | tachykinin 1 | 21333 |
| MH700 | Tacr1 | tachykinin receptor 1 | 21336 |
| MH589 | Tacr2 | tachykinin receptor 2 | 21337 |
| MH665 | Tacr3 | tachykinin receptor 3 | 21338 |
| ST62 | Tacstd1 | tumor-associated calcium signal transducer 1 | 17075 |
| MH499 | Tbr1 | T-box brain gene 1 | 21375 |
| MH809 | Tcf2 | transcription factor 2 | 21410 |
| MH770 | Tcf3 | transcription factor 3 | 21415 |
| MH934 | Tcf4 | transcription factor 4 | 21413 |
| MH771 | Tcf7 | transcription factor 7, T-cell specific | 21414 |
| ST79 | Tcf7 | transmembrane protein 2 | 83921 |
| HD19 | Tcf7l2 | transcription factor 7-like 2, T-cell specific, HMG-box | 21416 |
| MH627 | Tcfe2a | transcription factor E2a | 21423 |
| HD62 | Tctex1 | t-complex testis expressed 1 | 21648 |
| MH880 | Tegt | testis enhanced gene transcript | 110213 |
| MH500 | Tera | teratocarcinoma expressed, serine rich | 56306 |
| MY221 | Tff1 | trefoil factor 1 | 21784 |
| MY222 | Tff2 | trefoil factor 2 (spasmolytic protein 1) | 21785 |
| MY224 | Tff3 | trefoil factor 3, intestinal | 21786 |
| MH532 | Tgfb1i4 | transforming growth factor beta 1 induced transcript 4 | 21807 |
| MH935 | Tgfbr1 | transforming growth factor, beta receptor I | 21812 |
| MH1260 | Tgtp | T-cell specific GTPase | 21822 |
| MH130 | Th | tyrosine hydroxylase | 21823 |
| HD40 | Thap1 | THAP domain containing, apoptosis associated protein 1 | 73754 |
| MH881 | Thra | thyroid hormone receptor alpha | 21833 |
| MH501 | Thrsp | thyroid hormone responsive SPOT14 homolog (Rattus) | 21835 |
| MH1011 | Titf1 | thyroid transcription factor 1 | 21869 |
| MH424 | Tle1 | transducin-like enhancer of split 1, homolog of Drosophila E(spl) | 21885 |
| MH392 | Tle3 | transducin-like enhancer of split 3, homolog of Drosophila E(spl) | 21887 |
| ST56 | Tm7sf3 | transmembrane 7 superfamily member 3 | 67623 |
| HD31 | Tmeff1 | transmembrane protein with EGF-like and two follistatin-like domains 1 | 230157 |
| MY223 | Tmem1 | transmembrane protein 1 | 216131 |
| HD49 | Tmod3 | tropomodulin 3 | 50875 |
| MH666 | Tmpo | thymopoietin | 21917 |
| MY112 | Tmprss3 | transmembrane protease, serine 3 | 140765 |
| DC25 | Tnni3 | troponin I, cardiac | 21954 |
| HD43 | Tnrc6 | trinucleotide repeat containing 6 | 233833 |
| MH1100 | Tomm70a | translocase of outer mitochondrial membrane 70 homolog A (yeast) | 28185 |
| MH502 | Top2a | topoisomerase (DNA) II alpha | 21973 |
| MH936 | Top2b | topoisomerase (DNA) II beta | 21974 |
| DA122 | Traf4 | Tnf receptor associated factor 4 | 22032 |
| MH968 | Trhr | thyrotropin releasing hormone receptor | 22045 |
| ST84 | Trim25 | tripartite motif protein 25 | 217069 |
| MH882 | Trim27 | tripartite motif protein 27 | 19720 |
| MH533 | Trim28 | tripartite motif protein 28 | 21849 |
| ST85 | Trim9 | tripartite motif protein 9 | 94090 |
| MH504 | Trp53 | transformation related protein 53 | 22059 |
| MH667 | Trp53bp2 | transformation related protein 53 binding protein 2 | 209456 |
| MH587 | Trp53inp1 | transformation related protein 53 inducible nuclear protein 1 | 60599 |
| MH613 | Trpc4 | transient receptor potential cation channel, subfamily C, member 4 | 22066 |
| MY290 | Trpm2 | transient receptor potential cation channel, subfamily M, member 2 | 28240 |
| MH883 | Tsc2 | tuberous sclerosis 2 | 22084 |
| MY267 | Tsga2 | testis specific gene A2 | 22092 |
| MH385 | Ttc3 | tetratricopeptide repeat domain 3 | 22129 |
| MH947 | Ttyh1 | tweety homolog 1 (Drosophila) | 57776 |
| MH534 | Tubb4 | tubulin, beta 4 | 22153 |
| MY240 | Tulp4 | tubby like protein 4 | 68842 |
| DC32 | Txnip | thioredoxin interacting protein | 56338 |
| HD56 | Tyr | tyrosinase | 22173 |
| MY270 | U2af1 | U2 small nuclear ribonucleoprotein auxiliary factor (U2AF) 1 | 108121 |
| MH1250 | Ube2g2 | ubiquitin-conjugating enzyme E2G 2 | 22213 |
| MH134 | Ube3a | ubiquitin protein ligase E3A | 22215 |
| MH1013 | Uchl1 | ubiquitin carboxy-terminal hydrolase L1 | 22223 |
| DA76 | Uncx4.1 | Unc4.1 homeobox (C. elegans) | 22255 |
| MH628 | Usf1 | upstream transcription factor 1 | 22278 |
| MH884 | Usf2 | upstream transcription factor 2 | 22282 |
| MY268 | Usp16 | ubiquitin specific protease 16 | 74112 |
| MH386 | Usp21 | ubiquitin specific protease 21 | 30941 |
| MH717 | Usp48 | ubiquitin specific protease 48 | 170707 |
| DC45 | Vamp2 | vesicle-associated membrane protein 2 | 22318 |
| MH1039 | Vax1 | ventral anterior homeobox containing gene 1 | 22326 |
| DC33 | Vdac2 | voltage-dependent anion channel 2 | 22334 |
| MH810 | Vdr | vitamin D receptor | 22337 |
| MH885 | Viaat | vesicular inhibitory amino acid transporter | 22348 |
| MH937 | Vil2 | villin 2 | 22350 |
| MH505 | Vim | vimentin | 22352 |
| MH886 | Vip | vasoactive intestinal polypeptide | 22353 |
| MH939 | Vipr1 | vasoactive intestinal peptide receptor 1 | 22354 |
| MH938 | Vipr2 | vasoactive intestinal peptide receptor 2 | 22355 |
| MH1225 | Vldlr | very low density lipoprotein receptor | 22359 |
| MH152 | Vps18 | vacuolar protein sorting 18 (yeast) | 228545 |
| HD77 | Wdr18 | WD repeat domain 18 | 216156 |
| MY254 | Wdr4 | WD repeat domain 4 | 57773 |
| MY275 | Wdr9 | WD repeat domain 9 | 93871 |
| MH772 | Wif1 | Wnt inhibitory factor 1 | 24117 |
| MH590 | Wnt1 | wingless-related MMTV integration site 1 | 22408 |
| MH887 | Wnt10a | wingless related MMTV integration site 10a | 22409 |
| MH811 | Wnt10b | wingless related MMTV integration site 10b | 22410 |
| MH940 | Wnt11 | wingless-related MMTV integration site 11 | 22411 |
| MH941 | Wnt16 | wingless-related MMTV integration site 16 | 93735 |
| MH591 | Wnt2 | wingless-related MMTV integration site 2 | 22413 |
| MH592 | Wnt2b | wingless related MMTV integration site 2b | 22414 |
| MH593 | Wnt3 | wingless-related MMTV integration site 3 | 22415 |
| MH594 | Wnt3a | wingless-related MMTV integration site 3A | 22416 |
| MH595 | Wnt4 | wingless-related MMTV integration site 4 | 22417 |
| MH669 | Wnt5a | wingless-related MMTV integration site 5A | 22418 |
| MH942 | Wnt5b | wingless-related MMTV integration site 5B | 22419 |
| MH643 | Wnt6 | wingless-related MMTV integration site 6 | 22420 |
| MH644 | Wnt7a | wingless-related MMTV integration site 7A | 22421 |
| MH645 | Wnt7b | wingless-related MMTV integration site 7B | 22422 |
| MH773 | Wnt8a | wingless-related MMTV integration site 8A | 20890 |
| MH670 | Wnt8b | wingless related MMTV integration site 8b | 22423 |
| MH812 | Wnt9a | wingless-type MMTV integration site 9A | 216795 |
| MH943 | Wnt9b | wingless-type MMTV integration site 9B | 22412 |
| MH944 | Wt1 | Wilms tumor homolog | 22431 |
| MH1040 | Xbp1 | X-box binding protein 1 | 22433 |
| MH535 | Xpo1 | exportin 1, CRM1 homolog (yeast) | 103573 |
| MH1158 | Ythdc2 | YTH domain containing 2 | 240255 |
| MH536 | Ywhab | tyrosine 3-monooxygenase/tryptophan 5-monooxygenase activation protein, beta polypeptide | 54401 |
| MH537 | Ywhae | tyrosine 3-monooxygenase/tryptophan 5-monooxygenase activation protein, epsilon polypeptide | 22627 |
| MH888 | Ywhag | 3-monooxgenase/tryptophan 5-monooxgenase activation protein, gamma polypeptide | 22628 |
| MH538 | Ywhah | tyrosine 3-monooxygenase/tryptophan 5-monooxygenase activation protein, eta polypeptide | 22629 |
| MH506 | Ywhaq | tyrosine 3-monooxygenase/tryptophan 5-monooxygenase activation protein, theta polypeptide | 22630 |
| MH507 | Ywhaz | tyrosine 3-monooxygenase/tryptophan 5-monooxygenase activation protein, zeta polypeptide | 22631 |
| MH672 | Za20d2 | zinc finger, A20 domain containing 2 | 22682 |
| MH1251 | Zbtb16 | zinc finger and BTB domain containing 16 | 235320 |
| MH775 | Zbtb20 | zinc finger and BTB domain containing 20 | 56490 |
| HD28 | Zfhx1b | zinc finger homeobox 1b | 24136 |
| MH671 | Zfp101 | zinc finger protein 101 | 22643 |
| MH774 | Zfp161 | zinc finger protein 161 | 22666 |
| MH813 | Zfp238 | zinc finger protein 238 | 30928 |
| MH1226 | Zfp294 | zinc finger protein 294 | 78913 |
| MH1252 | Zfp295 | zinc finger protein 295 | 114565 |
| MH1235 | Zfp316 | zinc finger protein 316 | 54201 |
| MH464 | Zfp358 | Zink finger protein 358 | 140482 |
| MH1157 | Zfp423 | zinc finger protein 423 | 94187 |
| MH819 | Zfp503 | zinc finger protein 503 | 218820 |
|  |  |  |  |
