# Supplementary material for: Regulatory Pathway Analysis by High-Throughput In Situ Hybridization
Source: PLoS Genet. 2007 Oct 19;3(10):e178. doi: 10.1371/journal.pgen.0030178 (PMC2041993; doi:10.1371/journal.pgen.0030178)
Supplement: Supplementary file 3 [file pgen.0030178.st002.doc]

| Supplemental Table 2  Filtering of 1K ISH Data Set for Candidate Pax6 Target Genes | | | | | |
| --- | --- | --- | --- | --- | --- |
| **Filter 1**  **Genes with Expression Pattern overlapping with that of Pax6 (Custer 7)** | **Filter 2**  **Cluster 7 Genes Coexpressed with *Pax6* in Cortex** | **Filter 3**  **Genes whose Expression Pattern is Changed in Cortex of *Pax6****^sey/sey^* | **Filter 4**  **Pax6 Binding Site Predicted** | **Filter 5**  **EMSA Observed** | |
| *Zbtb20* | yes | no |  |  | |
| *Grm3* | no |  |  |  | |
| ***D930015E06Rik*** | **yes** | **yes** | **yes** | **shift but no supershift** | |
| *Igfbpl1* | yes | yes | no |  | |
| *Chrna4* | yes | no |  |  | |
| *Syt6* | no |  |  |  | |
| ***Lrp5*** | **yes** | **yes** | **yes** | **yes** | |
| ***Fzd2*** | **yes** | **yes** | **yes** | **shift but no supershift** | |
| *Arhgap5* | yes | no |  |  | |
| *Wnt7b* | no |  |  | |  |
| *Wnt7a* | yes | no |  | |  |
| *Grin1* | no |  |  | |  |
| *Rasgrf1* | no |  |  | |  |
| *Pnck* | no |  |  | |  |
| *Gabrb1* | yes | no |  | |  |
| *6330404F12Rik* | yes | no |  | |  |
| *Gria1* | no |  |  | |  |
| ***Bcan*** | **yes** | **yes** | **yes** | **shift but no supershift** | |
| *Gad2* | yes | no |  |  | |
| *Chrnb4* | no |  |  |  | |
| *Dtx1* | no |  |  |  | |
| *Cdh8* | no |  |  |  | |
| *Calb2* | no |  |  |  | |
| ***Neurod6*** | **yes** | **yes** | **yes** | **yes** | |
| ***Neurog2*** | **yes** | **yes** | **yes** | **yes** | |
| ***Neurod1*** | **yes** | **yes** | **yes** | **yes** | |
| *Lhx1* | no |  |  |  | |
| ***Pax6*** |  | **yes** | **yes** | **yes** | |
| *Tmem2* | no |  |  |  | |
| ***Odz2*** | **yes** | **yes** | **>100 sites** | **not tested** | |
| *Lhx2* | yes | no |  |  | |
| ***Trim9*** | **yes** | **yes** | **yes** | **yes** | |
| ***Pde1b*** | **yes** | **yes** | **yes** | **yes** | |
| ***Sst*** | **yes** | **yes** | **yes** | **previously identified target** | |
| *Ptprt* | no |  |  |  | |
| *Calb1* | no |  |  |  | |
| *Zfp423* | no |  |  |  | |
| *9130024F11Rik* | no |  |  |  | |
| *Adcy8* | no | no |  |  | |
| *Slc6a1* | no | no |  |  | |
| ***Arx*** | **yes** | **yes** | **yes** | **yes** | |
| *Tac1* | no |  |  |  | |
| *2900042E01Rik* | no |  |  |  | |
| *Avp* | no |  |  |  | |
| *Bdnf* | no |  |  |  | |
| *Cck* | no |  |  |  | |
| *Cdh13* | no |  |  |  | |
| *Cer1* | no |  |  |  | |
| *Cntf* | no |  |  |  | |
| *Efnb3* | no |  |  |  | |
| *Eomes (Tbr2)* | yes | yes | no |  | |
| *Fgf14* | no |  |  |  | |
| *Fgf15* | no |  |  |  | |
| *Fzd8* | yes | no |  |  | |
| *Gabrb2* | no |  |  |  | |
| *Gm623* | no |  |  |  | |
| *Kcnj6* | no |  |  |  | |
| *Grm1* | no |  |  |  | |
| *Lhx5* | no |  |  |  | |
| *Lhx9* | yes | no |  |  | |
| ***Neurod4*** | **yes** | **yes** | **yes** | **shift no supershift** | |
| ***Neurog1*** | **yes** | **yes** | **yes** | **yes** | |
| *Nr2e1 (Tlx)* | yes | no |  |  | |
| *Optn* | no |  |  |  | |
| *Otx1* | yes | no |  |  | |
| *Otx3* | no |  |  |  | |
| *Park2* | no |  |  |  | |
| *Pax7* | no |  |  |  | |
| *Pde1c* | no |  |  |  | |
| *Plxnc1* | no |  |  |  | |
| *Pou3f4* | no |  |  |  | |
| *Rax* | no |  |  |  | |
| *Rora* | no |  |  |  | |
| *Rorb* | yes | no |  |  | |
| *Sla* | no |  |  |  | |
| *Tcf7l2* | no |  |  |  | |
| *Ttyh1* | yes | no |  |  | |
| *Wnt1* | no |  |  |  | |
| *Wnt2b* | no |  |  |  | |
| *Wnt3* | no |  |  |  | |
| *Wnt3a* | no |  |  |  | |
|  | | | | | |

Genes that have passed all five filters are in **bold**
