# Supplementary material for: Regulatory Pathway Analysis by High-Throughput In Situ Hybridization
Source: PLoS Genet. 2007 Oct 19;3(10):e178. doi: 10.1371/journal.pgen.0030178 (PMC2041993; doi:10.1371/journal.pgen.0030178)
Supplement: Supplementary file 4 [file pgen.0030178.st003.doc]

Supplemental Table 3

DNA sequence of the actual Pax6 binding sites on the selected target genes

| **Gene-Site** | **Predicted site (mouse)** | **Binding** | **Supershift** |
| --- | --- | --- | --- |
| Arx-1 | ACAAACTCAGCCCTAAAAATA | no | no |
| Arx-2 | ACATGTCACGCATGACAAACT | yes | yes |
| Bcan-1 | CCTCTGTCGAGGAACCCCCAC | no | no |
| Bcan-2 | CCTCTGTCGAGGAACCCCCAC | yes | no |
| D930015E06Ril-1 | ACTTCTGGTAGTAACTGTCTG | no | no |
| D930015E06Ril-2 | TTAAACAAAGCTTAAACTAGG | no | no |
| D930015E06Ril-3 | CTGCTAACCTTGACCTCAATC | no | no |
| D930015E06Ril-4 | TAAACAGGAACTCAAAACCCG | no | no |
| D930015E06Ril-5 | TGCAGTTATTTCCTGCCTATT | yes | no |
| D930015E06Ril-6 | TTAAACAAAGCTTAAACTAGG | yes | no |
| D930015E06Ril-7 | ACTTCTGGTAGTAACTGTCTG | no | no |
| Fzd2 | CTTAAGTTACAGGAAAGAGAG | yes | no |
| Lrp5 | GTGCTGACCTGGGAACAGGAG | yes | no |
| Neurod1-1 | GGGCACTGAGTCATGAAAAGC | yes | yes |
| Neurod1-2 | ATTGTTTATGCATGAATGAAC | yes | yes |
| Neurod4 | TCTCATTTATCCTTGAAAATC | yes | no |
| Neurod6-1 | CAGCATTCATGCATCAACACA | yes | yes |
| Neurog1 | GGGCTTCATGCATTATGGATC | yes | yes |
| Neurog2 | GATAAATAATGCATGAAGCAA | yes | yes |
| Pax6-1 | CTGTTTGATGCTTGAATTCAC | yes | yes |
| Pax6-2 | TTCATTCTCGTCTGAGTGATC | no | no |
| Pax6-3 | TTGTTTTCCGCCTCACTGATT | yes | yes |
| Pax6-4 | ATTTTTAACGTGTAATTTTAA | no | no |
| Pdelb | TAGCTTCATGCTTCAGCTAAT | yes | yes |
| Trim9-1 | TGAAGTCATGCTTGAGTTATT | yes | yes |
| Trim9-2 | TATCATTGAATCTTAAATGTA | no | no |
| Pax6 consensus | ANNTTCACGCWTSANTKMNY | **-** | - |
| Positive | ACATTCACGCATGACTGACT | yes | yes |
| Sst | GCTTTTACGCACCATTAGCC | yes | - |
| E3.2 | TGCTTCATGCATTATTTATC | yes | - |
| E1.1 | TCATTCACGCCTAGAAGCAG | yes | - |
| mtE1.1 | TCACTAGTAACGAGAAGCAG | no | - |
