# Supplementary material for: Regulatory Pathway Analysis by High-Throughput In Situ Hybridization
Source: PLoS Genet. 2007 Oct 19;3(10):e178. doi: 10.1371/journal.pgen.0030178 (PMC2041993; doi:10.1371/journal.pgen.0030178)
Supplement: Supplementary file 5 [file pgen.0030178.st004.doc]

Supplemental Table 4.

Cluster 10 genes coexpressed with *Pax6* in the neocortex at E14.5^[1]^

| 0610016J10Rik | Gas5 |
| --- | --- |
| **Birc5** | Gmps |
| Catna1 | Gnai2 |
| Ccna2 | Kif11 |
| **Ccnd2** | Kif4 |
| Cdc2a | Kifc5a |
| Cdca1 | **Lrig1** |
| Chaf1b | Mdk |
| Col18a1 | Mif |
| Cpsf5 | Mki67 |
| **Cxcl12** | **Ndrg2** |
| E2f3 | Nudc |
| Efnb1 | Plxnb1 |
| Enc1 | Prim1 |
| Fgfr1 | **Sema5a** |
| **Fgfr2** | Sfrp2 |
| Fkbp3 | **Sod1** |
| **Foxm1** | Sox9 |
| G3bp | Top2a |
| **Gadd45g** | Vdac2 |
|  | Vim |

1. Genes marked in bold are differentially expressed in *Pax6^sey/sey^*
